# Effectiveness, structure, and content of nurse counseling in gynecologic oncology: a systematic review

**DOI:** 10.1186/s12912-017-0237-z

**Published:** 2017-08-03

**Authors:** Silvia Raphaelis, Andrea Kobleder, Hanna Mayer, Beate Senn

**Affiliations:** 1 Department of Nursing Science, University of Vienna, Alser Strasse 23, 1080 Vienna, Austria; 2 Institute for Applied Nursing Sciences IPW-FHS, University of Applied Sciences FHS St. Gallen, Rosenbergstrasse 59, 9001 St. Gallen, Switzerland; 3 Sydney Nursing School, The University of Sydney, Mallett Street 88, Camperdown, NSW 2050 Australia

**Keywords:** Systematic review, Oncology nursing, Female genital Neoplasms, Counseling, Patient education as topic

## Abstract

**Background:**

Gynecological pre-cancer and gynecological cancers are considerable diseases in women throughout the world. The disease and treatment lead to numerous biopsychosocial issues. To improve the outcomes of affected women, several counseling interventions have been tested thus far in nursing research. These interventions target different endpoints and are composed of various structural and content components. The purpose of this research was to systematically review the effectiveness of nurse counseling on any patient outcomes tested so far in gynecologic oncology before, during and after treatment and to explore structure and content components.

**Methods:**

Experimental, quasi-experimental, and pre-experimental studies assessing the effectiveness of nurse counseling in women with gynecological neoplasia were searched for in PubMed®, CINAHL®, PsychINFO®, Cochrane®, and EMBASE®. Reference lists were hand-searched and relevant authors were contacted. Moreover, the evidence level and methodological quality of the included studies were assessed. Afterwards, the effect of nurse counseling on each identified patient outcome was narratively analyzed. To identify the structural and content components of the included interventions, a structured content analysis was performed. Finally, it was determined which components were associated with favorable outcomes within the included studies.

**Results:**

Seven experimental and three pre-experimental studies, reporting the effects of 11 interventions on a total of 588 participants, were eligible. No study investigated women with pre-cancer. Three studies had a high, five a moderate, and two a low methodological quality. Positive effects were found on quality of life, symptoms, and healthcare utilization. Eight structural components and four content components composed of various sub-components were identified and linked to specific effects.

**Conclusions:**

The current evidence base is fragmented and inconsistent. More well-designed, large-scale studies including women with pre-cancer are warranted. Most convincing evidence indicates that nurse counseling can improve symptom distress. Components associated with the most trustworthy effects include nurses with an academic education; repeated and individual consultations during and after active treatment; structured, tailored, interdisciplinary orientated, and theoretically based counseling concepts; specific materials; comprehensive symptom management; and utilization of healthcare services. Healthcare providers and researchers can use the findings of this review for the systematic development of nurse counseling in gynecologic oncology.

**Electronic supplementary material:**

The online version of this article (doi:10.1186/s12912-017-0237-z) contains supplementary material, which is available to authorized users.

## Background

Women with gynecological neoplasia are affected by cellular changes in the female reproductive organs, such as the uterus, ovaries, cervix, vulva, and vagina. These cellular changes can be based on precancerous (e.g., cervical intraepithelial neoplasia) or cancerous (e.g., cervical cancer) neoplasia. They represent considerable diseases in women all over the world with an annual incidence of 1 to 2% for gynecological pre-cancer [1–3] and of 14.5 to 22.1% for gynecological cancer [4]. Especially if left untreated, some pre-cancers may develop into invasive cancer [5, 6]. Due to this linkage, some precancerous conditions (e.g., vulvar intraepithelial neoplasia grade III) are incorporated as carcinoma in situ [7, 8] in the international classification of specific gynecologic cancer forms. Pre-cancers are usually detected by abnormal clinical, cytological (e.g., Papanicolaou’s test), or human papillomavirus findings which require further examination by colposcopy and/or biopsy to gain a certain diagnosis [9, 10]. For gynecological cancer, various diagnostic procedures, from imaging techniques to histological examinations, are available. After diagnostic clarification, appropriate medical treatment (e.g., surgery) and care [3, 10, 11] is scheduled for women of both conditions, within the same cancer-specialized healthcare settings and performed by the same oncological healthcare providers [12–14]. The disease and treatment leads to similar biopsychosocial symptoms for women of both conditions [15], including pain, fatigue, and anxiety affecting quality of life [12, 13] and issues of women’s health, such as femininity, sexuality, and intimate relationships [16–18]. Qualitative studies, in particular, indicate that affected women need specific counseling regarding their concerns [19–22].

Several studies have so far evaluated the effect of nurse counseling in women with gynecological neoplasia. The study interventions are comprised of various structural and content components that cover a broad range in terms of the intervention provider, the time frame, the mode of delivery, and the content provided. Studies concerning pre-cancer have primarily investigated women who were scheduled for colposcopy to further diagnose preliminary findings (e.g., [23]). In women with gynecologic cancer, several intervention studies (e.g., [24–26]) and the systematic review by Cook et al. [27] are available. However, the systematic review focuses solely on the impact of specialized nurses on selected outcomes (quality of life, patient satisfaction, psychological outcomes) and in a brief section on specific structural components (time frame, mode of delivery).

Thus, the effectiveness of nurse counseling on different patient outcomes in women with gynecological neoplasia, as well as the structural and content components of these counseling interventions, have not been comprehensively reviewed to date. To inform future research and healthcare providers about the current evidence base in this area, this review aims to:

1. explore the impact of nurse counseling on different patient outcomes,
2. identify structure and content components of interventions tested so far, and
3. determine which components are associated with favorable outcomes.

## Methods

### Search and screening strategy

**Fig. 1:**
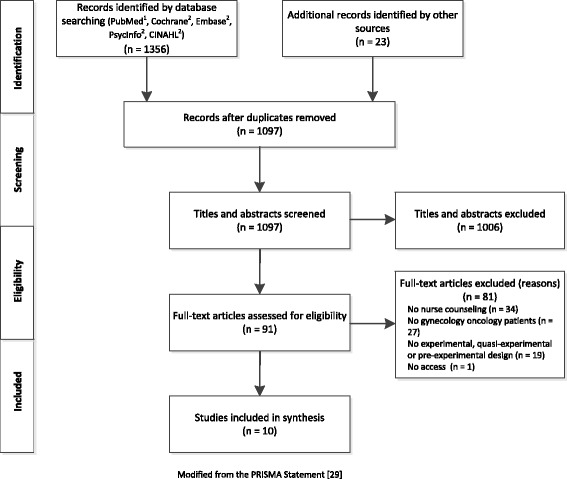
Flowchart of study selection. ^1^PubMed: #1: randomized controlled trial[pt] OR controlled clinical trial[pt] OR randomized[tiab] OR randomly[tiab] OR clinical trials as topic[MeSH: noexp] OR trial[tiab] OR groups[tiab]; #2: female genital neoplasms[MeSH]; #3: gyn?ecologi* OR Female genital OR ovar* OR fallopian tube OR uter* OR corpus OR endometri* OR cervi* OR vagin* OR vulv*; #4: cancer* OR carcinoma* OR sarcoma* OR malignan* OR neoplas* OR oncolog* OR tumor* OR tumour* OR adenocarcinoma* OR melanoma* OR dsyplas* OR papillomavirus infection [MeSH]: #5: 3 AND 4; #6: 2 OR 5; #7: nursing[MeSH] OR nurses[MeSH] OR nurs*; #8: counseling[MeSH] OR teaching materials[MeSH] OR patient education as topic[MeSH] OR self care[MeSH] OR education[MeSH] OR pamphlet[MeSH] OR [health promotion[MeSH] OR telephone[MeSH] OR counsel* OR “patient education” OR “self management” OR symptom management” OR “self care” OR inform* OR support* OR advice* OR consult* OR session* OR workshop* OR nurse management OR specialized OR specialized OR (psychosocial AND(intervention* OR support* OR program*)); #9: 8 OR 9; #10: 1 AND 6 AND 10. ^2^The PubMed search strategy (see above) was adjusted to the database interfaces of Cochrane, Ovid, and EBSCO

A comprehensive literature search [28, 29] was performed in PubMed®, CINAHL®, PsychINFO®, Cochrane®, and EMBASE® until July 2016. The search strategy (Fig. 1) was developed for PubMed and then adapted for all other databases. To identify additional studies, reference lists of relevant articles were hand-searched and authors contacted.

Inclusion criteria for retrieved records were English publications of experimental (randomized controlled trials), quasi-experimental (pretest-posttest studies and time series with non-equivalent comparison groups), or pre-experimental (one-group pretest-posttest studies and posttest-only studies with comparison groups) studies assessing the effectiveness of nurse counseling. Nurse counseling was defined as a problem-solving approach to support patients by a voluntary, interaction-intensive orientation, planning, and decision process. All outcomes of women with gynecological pre-cancer confirmed by colposcopy/biopsy (cervical, vulvar, or vaginal intraepithelial neoplasia) or with gynecological cancer (of the cervix, vulva, vagina, endometrium, ovary, or fallopian tube) were eligible. Interventions not mainly provided by a nurse and those designed for palliative patients, children, or adolescents were excluded.

### Study selection

After removing duplicates, two authors (SR and AK) independently screened the titles and abstracts of all records and, subsequently, the full-text versions of all remaining records for eligibility. They documented the inclusion and exclusion criteria for each study and discussed disagreements until consensus was reached.

### Assessment of study quality

Subsequently, the two authors independently assessed the methodological quality of included studies, whereby a statistician supported the appraisal of appropriate statistical analysis. Experimental studies were assessed with the Joanna Briggs Institute 2014 [30] (JBI) tool for *Randomized Control/Pseudo-randomized Trials* and for all other studies, the JBI tool for *Descriptive/Case Series Studies* was appropriate (Additional file 1). The sum quality score for each study was converted into percentage values and interpreted in accordance with studies based on similar methodologies (e.g., [31, 32]). Values from 0 to 49% were defined as of low methodological quality, and those from 50 to 79% and from 80 to 100% as of moderate and high methodological quality, respectively. The two authors compared their assessment results against one another and discussed any disagreement until consensus was reached. Finally the evidence level (Additional file 2) of each included study design was determined with the JBI classification [33] (from level 1, experimental designs, to level 4, observational-descriptive designs).

### Data extraction

After this assessment process, the first author extracted the characteristics and results of all included studies and contacted the study authors to gather any missing information in this regard.

### Methods of synthesis

For data synthesis, the effect of nurse counseling on each identified patient outcome was narratively analyzed regarding (1) direction of the effect, (2) consistency of the effect across studies, and (3) strength of the evidence for the effect (evidence level, study quality) in accordance with the systematic review methodology [28, 34]. To identify structural and content components, the study reports of included studies were analyzed with a structured content analysis [35, 36]. Identification of structural components was directed by the question of *how* nurse counseling was performed (who counseled whom, where, when, how often, how long, with what, and under which counseling concept?). In contrast, identification of content components was driven by the question of *what* themes were addressed during nurse counseling (what was counseled?).Throughout the process, the category system (main and sub-components of nurse counseling) was developed by a deductive-inductive strategy. Main components were developed by deduction from the above mentioned questions, whereas sub-components were formed by induction and deduction (by consultation of similar work (e.g., [27, 37, 38]) and literature-based conceptualizations (e.g., [39, 40]). Coding was performed at the level of paragraphs, phrases, and words by progressively summarizing the material. Data synthesis is presented by a description of the category system that emerged, the frequency of the components, and their association with effects on specific patient outcomes under consideration of the best available evidence (by evidence level and study quality).

## Results

### Summary of included studies

The literature search yielded 1356 records, from which ten studies could be included (Fig. 1). Seven investigations were experimental level 1 studies [25, 26, 41–45] and three were pre-experimental level 4 studies [46–48]. Three studies were of high [25, 43, 44], five were of moderate [26, 42, 45, 47, 48], and two were of low methodological quality [41, 46]. The interrater-reliability of the two authors who assessed the methodological quality was substantial (Cohen’s kappa = 0.84). Experimental trials suffered mostly from blinding of participants and an appropriate intention-to-treat approach. Pre-experimental trials mostly lacked strategies concerning confounding factors and an appropriate intention-to-treat approach (Additional file 3). The samples of three studies were considered as small, with fewer than 30 participants per study group [26, 42, 48] and therefore at risk for introducing type II errors. The studies by McCorkle et al. [43, 44] and by Donovan et al. [25] provide the best available evidence. Across all included studies, a total of 588 participants were investigated. Participants with various gynecological cancer sites were investigated most frequently [26, 41, 42, 45]. However, none of the studies included women with pre-cancer confirmed by colposcopy/biopsy. Across all included studies, 11 interventions were tested in total: the two reports of McCorkle et al. [43] and [44] address the same intervention, and each of the studies by Liu et al. [48] and Nolte et al. [45] examined two interventions. Two studies [45, 48] compared different counseling interventions against each other, while all others compared counseling with usual care. Of the ten authors contacted, six provided additional information. Study and intervention characteristics, effectiveness results, evidence level (thereinafter referred to as experimental or pre-experimental study), and study quality are presented in Table 1.

**Table 1:** Study and intervention characteristics

Reference & country	Study design	Participants	Interventions Structure components		Content components		Outcome measures Variables – Measures	Time points	Results	Study quality^a^	Evidence level^b^
Aktaş et al. 2014 [41] Turkey	RCT Repeated measures Experimental	Patients with gynecological cancer *N* = 70 *Intervention – Home Care Service: n* = 35 *Attention control: n* = 35 Age: *M* = 49 43% ovarian, 43% endometrial, 14% cervical cancer	Provider/receiver	Nurse (not specified)/patients	Disease	Psychosocial symptoms	Sexual satisfaction – *Golombok Rust Inventory of Sexual Satisfaction (GRISS)*	T_1_: pre-surgery T_2_: 12 weeks post-surgery	• Significant improvement in intervention group at T_2_ in overall sexual satisfaction (*p* = .001) and in its subscales ‘sexual non-communication’, ‘anorgasmia’, ‘avoidance’, ‘vaginismus’, ‘dissatisfaction’, and ‘non-sensuality’ (all *p* < .05) • No significant improvement in the ‘infrequency of sexual contact’ subscale (*p* = .77)	Low	1c
			Time frame	Repeated consultations before, during, and after active treatment	Treatment	Psychosocial symptoms					
			Mode of delivery	Face-to-face	Symptom Management	Symptom etiology; symptom prevention; symptom treatment					
			Format & setting	Individual counseling; clinic & home							
			Materials	Symptom-management guideline; individual care plans	Resources	Social network					
			Concepts	Structured & tailored counseling							
Chow et al. 2014 [42] China	RCT Mixed methods Feasibility study Repeated measures Experimental	Patients newly diagnosed with gynecological cancer scheduled for surgery *N* = 26 *Intervention – psychoeducational program*: *n* = 13 *Attention control: n* = 13 Age: *M* = 51 31% cervical, 54% uterine, 15% ovarian cancer	Provider/receiver	Non-APN with academic education/patients	Disease	Etiology; physical and psychosocial symptoms	Quality of life – *Traditional Chinese Functional Assessment of Cancer Therapy-General* Sexual functioning – *Sexual functioning-Vaginal changes Questionnaire* Uncertainty – *Mishel’s Uncertainty in Illness Scale* Anxiety/depression – *Hospital Anxiety & Depression Scale* Social support – *Medical Outcomes Study Social Support Survey*	T_1_: pre-surgery T_3_: 8 weeks post-surgery T_3_: 8 weeks post-surgery T_1_: pre-surgery T_2_: post-surgery T_3_: 8 weeks post-surgery	• No significant group differences in overall quality of life and its ‘physical’, ‘functional’, ‘emotional’ and ‘social’ subscales, in sexual functioning, in overall uncertainty and its ‘ambiguity’, ‘complexity’, and ‘unpredictability’ subscales, in anxiety and depression, and in social support (all *p* > .05) • Significant improvement at T_2_ in intervention group in the uncertainty subscale ‘inconsistency’ (*p* = .026)	Moderate	1c
			Time frame	Repeated, long consultations before, during, and after active treatment	Treatment	Therapeutic procedures; physical and psychosocial symptoms					
			Mode of delivery	Face-to-face & phone	Symptom Management	Symptom prevention; symptom treatment					
			Format & setting	Individual & group counseling; clinic							
			Materials	no materials	Resources	Personal capabilities; social network; healthcare services					
			Concepts	Structured & tailored; theoretical basis							
Cox et al. 2008 [46] United Kingdom	One-group pretest-posttest Case series Pre-experimental	Patients with ovarian cancer having completed chemotherapy *Telephone follow-up on chemotherapy symptom management: N* = 56 Age: *M* = 62	Provider/receiver	APN with academic education/patients	Disease	Etiology; diagnostic procedures	Quality of life – *FACT Ovarian (FACT O)* Patient experience and satisfaction –*self-designed patient experience and satisfaction questionnaire*	T_1_: after chemotherapy completion T_2_: 10 months from baseline	• No significant improvement in overall quality of life and its ‘physical’, ‘functional’, ‘social’, and ‘symptoms’ subscales (all *p* > .05) • Significant improvement in the ‘emotional’ quality of life subscale (*p* = .016) • Patient satisfaction and experience high (*M* = 8.24, *SD* = 2.0) at T_2_ (no pretest scores provided)	Low	4c
			Time frame	Repeated, short consultations after active treatment	Treatment	Physical and psychosocial symptoms					
			Mode of delivery	Phone	Symptom Management	Symptom etiology; symptom treatment					
			Format & setting	Individual counseling							
			Materials	Symptom-assessment tool; symptom-management guideline; leaflets	Resources	Personal capabilities; social network; healthcare services					
			Concepts	Structured & tailored counseling; inter-disciplinary orientation							
Donovan et al. 2014 [25] USA	RCT Waitlist-control Pilot study Repeated measures Experimental	Patients with persistent or recurrent ovarian cancer *N* = 65 *Intervention – Web-based symptom management: n* = 33 *Control: n* = 32 Age: *M* = 56	Provider/receiver	Non-APN with academic education/patients	Disease	Physical and psychosocial symptoms	Symptom outcomes – *Symptom Representation Questionnaire (SQR)*	T_1_: pre-intervention T_2_: 2 weeks post-intervention T_3_: 6 weeks post-intervention	• Significant improvement of symptom distress over time (*p* = .037) and of symptom severity at T_1_ in intervention group (*p* = .058) • No significant group differences in symptom consequences or symptom controllability (all *p* > .05) • Patients highly satisfied with intervention and web-based delivery (*Mdn* not provided)	High	1c
			Time frame	Repeated consultations before, during, and after active treatment	Treatment	Physical and psychosocial symptoms					
			Mode of delivery	Internet	Symptom Management	Symptom etiology; symptom assessment; goal setting & planning; symptom prevention/treatment; evaluation & modification					
			Format & setting	Individual counseling			Satisfaction with intervention – *self-designed questionnaire*	T_3_: 6 weeks post-intervention			
			Materials	Symptom-assessment tool; symptom-management guidelines; individual care plans							
			Concepts	Structured & tailored; interdisciplinary orien-tation theoretical basis;	Resources	Personal capabilities; healthcare services					
Liu et al. 2001 [48] Taiwan	Posttest-only with nonequivalent comparison group Observational-descriptive study Repeated measures Pre-experimental	Patients with cervical cancer who had radical hysterectomy *N* = 20 *Intervention I – Educational program for lower urinary tract self-care by a head nurse + written material*: *n* = 11 *Intervention II – Educational program for lower urinary tract self-care by a staff nurse*: *n* = 9	Provider/receiver	Intervention I & II: non-APN without academic education/patients	Disease	Intervention I & II: no content	Knowledge about Foley care & bladder training – *self-designed questionnaire*	T_1_: at discharge T_2_: at readmission 2 weeks later	• Significant improvement of knowledge in intervention I at T_1_ (*p* = .004), but not at T_2_ (*p* = .71) • No significant group differences at T_2_ in performance at home, urinalysis, or urine culture (all *p* > .05)	Moderate	4b
			Time frame	Intervention I & II: Repeated consultations during active treatment	Treatment	Intervention I & II: Physical symptoms					
			Mode of delivery	Intervention I & II: face-to-face	Symptom Management	Intervention I & II: symptom assessment; symptom prevention; symptom treatment; evaluation & modif	Home performance of Foley catheter self-care – *self-designed questionnaire* Urinalysis, urine culture – *laboratory records*	T_2_: at readmission 2 weeks later			
			Format & setting	Intervention I & II: individual; clinic							
			Materials	Intervention I: leaflet Intervention II: none							
			Concepts	Intervention I & II: structured counseling	Resources	Intervention I & II: no content					
Maughan et al. 2001 [26] United Kingdom	RCT Mixed methods Repeated measures Experimental	Patients with gynecological cancer and major pelvic surgery *N* = 36 *Intervention – Clinical Nurse Specialist intervention: n* = 19 *Control: n* = 17 Age: *M* = 50	Provider/receiver	APN with academic education/patients, families	Disease	Not specified	Quality of life – *European Organization for Research and Treatment of Cancer Quality of Life Questionnaire (EORTC QLQ-C30)*	T_1_: pre-surgery T_2_: 6 weeks post-surgery T_3_: 12 weeks post-surgery T_4_: 14 weeks post-surgery T_5_: 24 weeks post-surgery	• Significant improvement in overall quality of life (*p* = .04) in the intervention group • No significant group differences in the ‘physical’, ‘cognitive’, ‘emotional’, ‘social’, and ‘sexual’ quality of life subscales, as well as in sexual functioning (all *p* > .05)	Moderate	1c
			Time frame	Repeated consultations before, during, and after active treatment	Treatment	Therapeutic procedures; psycho-social symptoms					
			Mode of delivery	Face-to-face	Symptom Management	Symptom etiology; symptom prevention; symptom treatment					
			Format & setting	Individual counseling; clinic & home							
			Materials	Leaflets	Resources	Personal capabilities; social network; healthcare services	Sexual functioning – *Lasry Sexual Function Scale*	T_3_: 12 weeks post-surgery T_5_: 24 weeks post-surgery			
			Concepts	structured & tailored; interdisciplinary orientation							
McCorkle et al. 2009 [43] USA	RCT Repeated measures Experimental	Patients with ovarian cancer following surgery and scheduled for chemotherapy *N* = 149 *Intervention – Advanced Practice Nurse intervention + Psychiatric Consultation-Liaison Nurse (PCLN) for women with high distress: n* = 74 *Attention control: n* = 75 Age: *M* = 60	Provider/receiver	APN with academic education/patients, families	Disease	Physical & psychosocial symptoms	Depression – *Center for Epidemiological Studies-Depression Scale (CES-D)* Uncertainty – *Ambiguity subscale of the Mishel Uncertainty in Illness Scale (MUIS)* Symptom distress – *Symptom Distress Scale (SDS)* Overall quality of life – *Short-Form Health Survey (SF-12)*	T_1_: 24-48 h post-surgery T_2_: 1 month post-surgery T_3_: 3 months post-surgery T_4_: 6 months post-surgery	• Significant improvement of uncertainty concerning ambiguity (*p* = .018), symptom distress (*p* < .001), ‘physical’ and ‘cognitive’ quality of life subscales (all *p* < .001) over time in intervention group • No significant group differences over time in overall quality of life and depression (all *p* > .05)	High	1c
			Time frame	Repeated consultations during and after active treatment	Treatment	Decision-making; physical & psycho-social symptoms					
			Mode of delivery	Face-to-face & phone	Symptom Management	Symptom etiology; symptom assessment; goal setting & planning; symptom prevention, symptom treatment; evaluation & modification of strategies					
			Format & setting	Individual counseling; clinic & home							
			Materials	Symptom-assessment tool; symptom-management guideline; individual care plan							
			Concepts	Structured & tailored counseling; interdisciplinary orientation; theoretical basis	Resources	Social network; healthcare services					
McCorkle et al. 2011 [44] USA	Same study like McCorkle et al. 2009 [43]	*N* = 149 Intervention: *n* = 74 Attention control: *n* = 75 Age: *M* = 61	Same structure and content like in McCorkle et al. 2009 [43]				Healthcare utilization – *self-designed patient questionnaire, review of medical records*	T_1_: pre-surgery T_2_: 6 weeks post-surgery	• Significant less primary care visits in intervention group (*p* < .001) • No significant group differences in hospitalizations, oncology outpatient visits, and emergency room visits (all *p* > .05)	High	1c
Nolte et al. 2006 [45] USA	RCT Mixed methods Repeated measures Multicenter Experimental	Patients with gynecologic cancer and chemotherapy-induced alopecia *N* = 136 *Intervention I – standard counseling: n* = 68 *Intervention II – standard counseling + videotape*: *n* = 68 Age: *M* = 58 Ovarian, uterine, and cervical cancer	Provider/receiver	Intervention I & II: Nurse (not specified)/patients	Disease	Intervention I & II: no content	Body image & self-esteem – *Body Cathexis/Self-Cathexis Scale (BCSCS)*	T_1_: before chemotherapy cycle 1 T_2_: before chemotherapy cycle 3 T_3_: after chemotherapy cycle 4	• No significant group differences in body image and self-esteem (all *p* > .05)	Moderate	1c
			Time frame	Intervention I & II: One-time consultation before active treatment	Treatment	Intervention I & II: physical symptoms					
			Mode of delivery	Intervention I & II: face-to-face	Symptom Management	Intervention I & II: symptom etiology; symptom treatment					
			Format & setting	Intervention I & II: individual counseling; clinic							
			Materials	Intervention I & II: Symptom-management guideline Intervention II: video	Resources	Intervention I & II: healthcare services					
			Concepts	Intervention I & II: structured counseling; theoretical basis							
So et al. 2006 [47] China	One-group pretest-posttest Case series –	Patients with cervical cancer receiving brachytherapy	Provider/receiver	Nurse (not specified)/patients, families	Disease	No content	Knowledge & attitudes regarding vaginal douching – *self-designed* *questionnaire*	T_1_: presumably 1–2 weeks before brachytherapy T_2_: presumably 1–2 weeks before brachytherapy T_3_: admission day brachytherapy	• Significant improvement of knowledge from pre-intervention to T_2_ and from pre-intervention to T_3_ (*p* < .001) • Significant improvement of attitudes towards self-care from pre-intervention to T_2_ (*p* < .001), but not from pre-intervention to T_3_ (*p* > .05)	Moderate	4c
					Treatment	Physical symptoms					
	repeated measures Pre-experimental	*Education program on vaginal douching: N* = 30	Time frame	One-time, long consultation before active treatment	Symptom Management	Symptom etiology; symptom assessment, symptom prevention; symptom treatment					
			Mode of delivery	Face-to-face							
			Format & setting	Individual counseling (presumably); clinic							
			Concepts	Structured & tailored counseling	Resources	No content					

*RCT* randomized controlled trial, *M mean*, *p p*-value

^a^Low methodological quality refers to total quality scores ranging from 0 to 49%; Moderate methodological quality refers to total quality scores ranging from 50 to 79%; high methodological quality refers to total quality scores ranging from 80 to 100%

^b^Level 1 refers to experimental designs, level 2 to quasi-experimental designs, level 3 to observational-analytic designs, level 4 to observational-descriptive studies, and level 5 to expert opinion and bench research [33]

### Effects of nurse counseling

The effectiveness of nurse counseling is described in four main outcome groups: quality of life, symptoms, self-care performance, and healthcare utilization. The outcome group ‘quality of life’ refers to all reported effects of nurse counseling on quality of life total scores (such as overall quality of life) and quality of life sub-dimensions (such as cognitive or emotional quality of life dimensions) as measured and defined by the quality of life questionnaires used within the included studies. In distinction to the ‘symptom’ outcome group it is noteworthy that some quality of life measures used in the included studies contain questions about the occurrence of specific symptoms (e.g., [26]). For data synthesis it was not feasible to reconstruct which effects on symptoms were observed in these quality of life measures and to subsume them under the ‘symptom’ outcome group since studies did not report findings of single items. The outcome group ‘symptoms’ includes all reported effects of nurse counseling on physical and psychosocial symptoms outside quality of life measures as well as on the symptom severity, symptom distress, symptom consequences and symptom controllability of physical and psychosocial symptoms in general. In the outcome group ‘self-care performance’ only the performance of patients regarding physical symptoms was described in the included studies. Finally, the outcome group ‘healthcare performance’ refers to all reported effects of nurse counseling on the number of hospitalizations, oncology outpatient visits, emergency room visits, and primary care visits. The results are separated in each of these groups by experimental and pre-experimental studies.

#### Quality of life

In none of the experimental studies ‘functional’ [42], ‘emotional’ [26, 42], ‘social’ [26, 42], or ‘sexual’ [26] quality of life dimensions improved significantly with nurse counseling (Additional file 4). These results are based on moderate study quality [26, 42]. Contradictory results appeared in overall quality of life, as well as in the ‘physical’ and ‘cognitive’ aspects. Two studies with moderate quality observed significant positive effects on overall quality of life [26, 42], but not on the ‘physical’ and ‘cognitive’ dimensions. Conversely, the study by McCorkle et al. [43], with high methodological quality, reported no significant effects on overall quality of life, but did report effects on the ‘physical’ and ‘cognitive’ dimensions. Like all other studies, the pre-experimental study by Cox et al. [46], with low methodological quality, found no significant improvement of the ‘social’ and ‘functional’ aspects; similar to some of the other studies, it observed no effects on overall quality of life [43] and ‘physical’ aspects [26, 42], and, however, it was the only study that found significant improvements in ‘emotional’ quality of life dimensions.

#### Symptoms

Physical symptoms were measured in one pre-experimental study. The study was of moderate quality and compared two interventions against one another [48]. Thereby, counseling by a head nurse plus the provision of written information reduced urinary tract infection not significantly more than counseling by a staff nurse [48].

Psychosocial symptoms and issues were examined in seven research projects. In experimental investigations, no significant effects on anxiety [42], depression [42, 43], sexual functioning [26, 42], social support [42], overall uncertainty, and the uncertainty subscales of ‘complexity’ and ‘ambiguity’ [42] were found. Only the study that examined depression [43] was of high quality, whereas all others were of moderate methodological quality [26, 42]. In contrast, sexual satisfaction [41] and the uncertainty subscale of ‘inconsistency’ [42] improved significantly with nurse counseling. However, none of these results are based on studies with high methodological quality. Contradictory results appeared in the uncertainty subscale of ‘ambiguity’. One high-quality study observed positive effects on this outcome [43], whereas another moderate-quality study did not [42]. In another moderate-quality study counseling plus a video tape improved body image and self-esteem not significantly more than standard counseling [45]. When considering pre-experimental investigations, positive effects appeared in attitude and knowledge regarding vaginal douching [47]. Moreover, knowledge about urinary catheter care improved significantly with counseling by a head nurse plus written information when compared with counseling by a staff nurse [48]. However, both studies are of low quality.

Moreover, two experimental studies with high methodological quality demonstrated positive effects of counseling on symptom distress [25, 43]. One of these studies additionally investigated symptom severity, symptom consequences, and symptom controllability [25], although only symptom severity improved significantly with nurse counseling. The results of these studies relate to physical and psychosocial symptoms, but are not distinguishable on this level.

#### Self-care performance

In the moderate-quality pre-experimental study by Liu et al. [48], counseling by a head nurse plus the provision of written information improved urinary self-care performance not significantly more than counseling by a staff nurse.

#### Healthcare utilization

In one experimental study [44] investigating healthcare utilization nurse counseling yielded significantly fewer primary care visits, but no significant group differences in the number of hospitalizations, oncology outpatient visits, or emergency room visits.

### Structural components of nurse counseling

In total, eight main components with various sub-components characterizing the structure of nurse counseling in gynecologic oncology were identified (see Table 2 that includes a definition for each coded component).

**Table 2:** Structure and content components of nurse counseling in gynecologic oncology

Structural main components	Structural sub-components	Studies containing each coded component	Components with the best available evidence
Provider^a^	Non-APN without academic education	[48]	
	Non-APN with academic education	[25, 42]	✓
	APN with academic education	[26, 43, 44, 46]	✓
Receiver^b^	Patients	[25, 26, 41–48]	✓
	Families	[26, 43, 44, 47]	✓
Time frame^c^	Before active treatment	[45, 47]	
	During active treatment	[48]	
	After active treatment	[46]	
	During and after active treatment	[43, 44]	✓
	Before, during, and after active treatment	[25, 26, 41, 42]	✓
	One-time consultation	[45, 47]	
	Repeated consultation	[25, 26, 41–44, 46, 48]	
	Short consultation	[46]	
	Long consultation	[42, 47]	
Mode of delivery^d^	Face-to-face	[26, 41, 45, 47, 48]	
	Phone	[46]	
	Internet	[25]	✓
	Face-to-face and phone	[42–44]	✓
Format^e^	Individual counseling	[25, 26, 41, 43–48]	✓
	Individual and group counseling	[42]	
Setting^f^	Clinic	[42, 45, 47, 48]	
	Clinic and home	[26, 41, 43, 44]	✓
Materials	Symptom-assessment tools^g^	[25, 43, 44, 46]	
	Symptom-management guidelines^h^	[25, 41, 43–46]	
	Individual care plans^i^	[25, 41, 43, 44]	
	Leaflets^j^	[26, 46–48]	
	Videos^k^	[45, 47]	
Concepts	Structured counseling^l^	[45, 48]	
	Structured and tailored counseling^m^	[25, 26, 41–44, 46, 47]	✓
	Interdisciplinary orientation^n^	[25, 26, 43, 44, 46]	
	Theoretical basis^o^	[25, 42–45]	
Content main components	Content sub-components	Studies containing each coded component	Components with the best available evidence
Disease	Etiology^p^	[42, 46]	
	Diagnostic procedures^q^	[46]	
	Physical symptoms^r^	[25, 42–44, 46]	✓
	Psychosocial symptoms^s^	[25, 41–44, 46]	✓
Treatment	Therapeutic procedures^t^	[26, 42]	
	Decision-making^u^	[43, 44]	✓
	Physical symptoms^v^	[25, 42–48]	✓
	Psychosocial symptoms^w^	[25, 26, 41–44, 46]	✓
Symptom-management^x^	Symptom etiology	[25, 26, 41, 43–47]	✓
	Symptom assessment	[25, 43, 44, 47, 48]	✓
	Goal-setting and planning	[25, 43, 44]	✓
	Symptom prevention	[25, 26, 41–44, 47, 48]	✓
	Symptom treatment	[25, 26, 41–48]	✓
	Evaluation and modification of symptom management strategies	[25, 43, 44, 48]	✓
Resources^y^	Personal capabilities	[25, 26, 42, 46]	✓
	Social network	[26, 41–44, 46]	✓
	Healthcare services	[25, 26, 42–46]	✓

^a^The intervention provider, including specifications and educational background

^b^Patients and families (partners, parents etc.) as intervention receivers

^c^The time points (before/during/after active treatment), frequency (one-time/repeated counseling), and duration of each counseling session (sessions up to 20 min were considered as short consultations and sessions exceeding these frames as long consultations)

^d^The interaction channel by which interventions are delivered to recipients

^e^Individual counseling is provided to patients and families, whereas group counseling is delivered to a group of patients

^f^The counseling location, including inpatient and outpatient clinics and patients’ homes

^g^Any tools utilized by nurses or patients for symptom assessment

^h^Standardized recommendations regarding symptom self-care

^i^Any nursing plans regarding the care of individual patients

^j^Booklets by official organizations (such as Cancer Aid) and information sheets prepared by healthcare providers

^k^Videotapes shown or given to patients

^l^The intervention is delivered the same way for all recipients

^m^The intervention is adapted to the individual needs, priorities, and meanings of recipients

^n^Counselors consider interdisciplinary requirements to solve patients’ problems, such as collaborative and coordinative activities

^o^Any theoretical foundation of the counseling program (e.g., Orem’s self-care theory)

^p^Causes of gynecological neoplasia

^q^Any procedures used to diagnose gynecological neoplasia, including a discussion of diagnostic results

^r^Disease-related physical symptoms

^s^Disease-related psychosocial symptoms

^t^Any medical therapy for gynecological neoplasia, such as surgery, chemotherapy, or radiotherapy

^u^Patients’ decisions affecting subsequent treatment

^v^Treatment-related physical symptoms

^w^Treatment-related psychosocial symptoms

^x^The management of disease and treatment-related symptoms, including their causes, their assessment, goal-setting and subsequent planning of management strategies, prevention and treatment, as well as evaluation and modification of management strategies

^y^Any resources of patients to cope with disease and treatment-related issues, including personal capabilities (such as personal strengths, financial resources), the social network (utilization and communication), and healthcare services (utilization and communication)

#### Provider and receiver

Of the studies reporting sufficient details, two interventions were provided by a non-Advanced Practice Nurse (APN) without academic education [48], two interventions were by a non-APN with academic education [25, 42], and three interventions were implemented by an APN with academic education [26, 43, 44, 46]. Three interventions were offered to women’s families in addition to the women themselves [26, 43, 44, 47].

#### Time frame

The time points of intervention delivery ranged from the time before, during, and after active treatment. Two interventions were offered as one-time consultations [45, 47] and eight as repeated consultations [25, 26, 41–44, 46, 48]. Of the studies reporting information about the duration of each counseling session, one intervention can be characterized as short consultation [46] and two interventions as long consultations [42, 47].

#### Mode of delivery, format, and setting

Seven interventions were delivered face-to-face [26, 41, 45, 47, 48], one was delivered by phone [46], and one was delivered by internet [25]. In two interventions, face-to-face counseling was combined with counseling by phone [42–44]. Ten interventions employed an individual format for counseling [25, 26, 41, 43–48], and one combined individual counseling with group counseling [42]. Six interventions were offered in a clinical setting [42, 45, 47, 48], and three interventions were provided additionally at home [26, 41, 43, 44].

#### Materials

Symptom-assessment tools [25, 43, 44, 46] were used in three interventions, symptom-management guidelines in six [25, 41, 43–46], individual care plans in three [25, 41, 43, 44], leaflets in four [26, 46–48], and videos in two interventions [45, 47].

#### Concepts

Seven interventions were structured and tailored [25, 26, 41–44, 46, 47], and four interventions were structured only [45, 48]. Furthermore, four interventions had an interdisciplinary orientation towards solving patients’ problems [25, 26, 43, 44, 46], whereas an explicit theoretical basis was described for five interventions (e.g., Orem’s Self-Care Theory) [25, 42–45].

Structural components associated with significant improvements of outcomes in the studies with the best available evidence [25, 43, 44] included counseling by a nurse with an academic education providing repeated, individual consultations during and after active treatment in a structured, tailored, interdisciplinary orientated, and theoretically based manner. Furthermore symptom assessment tools, symptom-management guidelines, and/or individual care plans were utilized in these interventions. The components face-to-face counseling combined with phone counseling [43, 44], internet counseling [25], counseling of women’s families [43, 44], consultations in the clinic combined with consultations at home [43, 44], and counseling before active treatment [25] were each time applied in just one of the interventions with the most trustworthy results. Consequently, the presence as well as the absence of these components was associated with specific benefits. All other components were primarily associated with non-significant improvements when taking into account for each component the best available evidence (Additional file 5).

### Content components of nurse counseling

Data synthesis yielded four main components with different sub-components representing the themes discussed during nurse counseling (Table 2).

#### Disease and treatment

Counseling on issues pertinent to disease or treatment focused on disease etiology in two interventions [42, 46], on diagnostic or treatment-related procedures in three interventions [26, 42, 46], and on treatment-related decision-making in one intervention [43, 44]. Physical symptoms were addressed in nine [25, 42–48] and psychosocial symptoms in six interventions [25, 26, 41–44, 46].

#### Symptom management

Eight interventions focused on symptom etiology [25, 26, 41, 43–47], five on symptom-assessment [25, 43, 44, 47, 48], two on goal setting and planning [25, 43, 44], 11 on symptom prevention and/or treatment [25, 26, 41–48], and four on evaluation and modification of symptom-management strategies [25, 43, 44, 48].

#### Resources

How to utilize personal capabilities as resources to cope with disease and treatment-related issues was discussed in four interventions [25, 26, 42, 46]. Utilization of the social network was addressed in five [26, 41–44, 46] and utilization of healthcare services in seven interventions [25, 26, 42–46].

Content components intertwined with significant improvements of outcomes in the studies providing the best available evidence [25, 43, 44], included comprehensive counseling on the management of disease and treatment-related physical and psychosocial symptoms (from symptom etiology and symptom-assessment to the evaluation and modification of symptom-management strategies), as well as advice on the utilization of healthcare services. However, both the inclusion and exclusion of issues related to treatment-related decision-making [43, 44], utilization of personal capabilities [25], and utilization of the social network [43, 44] were associated with significant positive effects in these studies. All other components were primarily associated with non-significant improvements when taking into account for each component the best available evidence (Additional file 5).

## Discussion

This systematic review included ten studies, which tested 11 interventions in total. The results are limited to women with gynecologic cancer since none of the studies investigated women with pre-cancer confirmed by colposcopy/biopsy. One reason for this could be that focusing solely on rigorously confirmed conditions may complicate recruitment and result in smaller samples, as researchers need to review the diagnostic findings of potential participants. More than half of the studies represent pre-experimental investigations, studies with low methodological quality and/or pilot studies with small samples [25, 26, 42, 46–48]. Thus, when considering level of evidence, methodological quality, and replicated findings, the most robust results indicate that nurse counseling can improve symptom distress [25, 43], but not depression [42, 43]. These results are replicated in at least one experimental study with high quality. Moreover, eight structural components and four content components of nurse counseling with different sub-components were identified and examined on their association with positive effects.

### Effects of nurse counseling

The quality of life results show less unequivocal evidence of good quality. The results that were replicated across studies (e.g., functional quality of life) are supported by weak evidence [26, 42, 46] and those with stronger evidence (e.g., overall quality of life) [43] are contradictory [26, 42]. The contradictory findings may be due to different quality of life measures used across studies. Some measures inquired about symptom occurrence (e.g., [26]), whereas others focused on the impact of symptoms on quality of life (e.g., [43]). Additionally, overall quality of life is based on single items in some measures (e.g., [26]) and on total scores in others (e.g., [42]). Another issue is that many study reports do not describe how an intervention may yield intended quality of life effects. Thus, few studies may have selected the most proximal measures of cause-effect relations. However, other studies of breast cancer patients also observed inconsistent effects of nurse counseling on quality of life [49–51].

One study [48] showed that physical symptoms do not improve with counseling plus an add-on (leaflet, nurses’ position) when compared to standard counseling. Although this result is lacking strong evidence, it is reasonable that other factors contribute to the benefits of counseling. For example, previous research showed rather unfavorable results for the provision of written information alone [52]. The same applies to self-care performance, which also did not improve with counseling plus an add-on [48]. Overall, it is remarkable that only one study measured physical symptoms, although many interventions focused on their management [42, 43, 45–48]. It is possible that these studies evaluated them within quality of life [42, 43, 46] and symptom severity [25] measures. For psychosocial issues, it is hardly possible to draw any firm conclusions since the evidence for them is almost always weak. Most robust results indicate that nurse counseling cannot improve depression [42, 43]. This may be because that depression is a state or a personality trait [53]. A non-psychotherapeutic intervention such as nurse counseling should primarily improve depression as a state. The measures of included studies [42, 43] do not make this distinction, and thus, possibly failed to show depression state changes. However, in other studies with breast cancer patients, depression also remained unchanged with nurse counseling [49, 50, 54]. The reported improvements of symptom severity and distress [25, 43], which relate to physical and psychosocial symptoms and rely on strong evidence, indicate that nurse counseling has the potential to impact biopsychosocial symptoms. Particularly regarding symptom distress, two studies, one that employed an APN intervention [43] and one that tested a web-based intervention [25], came to the same result. This promising result was also found in previous nursing research with breast cancer patients [55].

In addition, nurse counseling seems to have few effects on healthcare utilization [44]. McCorkle et al. [44] argue that their APN intervention reduced primary care visits because it addressed women’s everyday concerns, whereas emergency room visits remained unchanged because patients were encouraged to seek urgent care if necessary. Conversely, hospitalizations and oncology outpatient visits were scheduled follow-ups, which in cancer care rely on well-established, pre-defined protocols and were thus not preventable. In contrast, previous research showed reduced emergency room and hospital visits due to oncology nursing [56].

### Structural components of nurse counseling

At least two-thirds of the interventions were implemented repeatedly [25, 26, 41–46, 48], face-to-face [26, 41–45, 47, 48], in an individual format [25, 26, 41, 43–48], in the clinical setting [25, 26, 41–48] with timely proximity to active treatment [25, 26, 41–45, 47, 48] and thereby involved structured and tailored counseling [25, 26, 41–44, 46, 47]. These components were also very common in previous work [27, 57] and are probably seen as part of a feasible, sustainable intervention structure. Around active treatment, patients have many clinical appointments, illness-related concerns, and self-care needs [19, 20, 22]. Their situation is mostly too complex and fluctuates too much for one consultation, and in the clinic, nurses can address patients’ questions after physician appointments. Of these rather frequently used components, repeated, individual consultations offered during and after active treatment, and delivered as both structured and tailored interventions, yielded the most robust improvements [25, 43, 44].

In contrast, few interventions involved home [26, 41, 43, 44], group [42], phone [42–44, 46], or internet [25] counseling, additional family members [26, 43, 44, 47], or symptom assessment tools. The common absence of symptom assessment tools is noteworthy, since many interventions focused on symptom management [25, 26, 41–48]. However, many of these components (e.g., phone counseling) can bridge critical transitions (e.g., discharge), between the clinical and home settings, but some might be difficult to implement (e.g., home counseling). Remarkably, these components were most often present in interventions with academically educated nurses [25, 26, 42–44, 46], who are probably more familiar with sophisticated, innovative approaches and roles. In this context, it must be emphasized that this provider specification, as well as the utilization of symptom assessment tools [25, 43, 44], were among those components that were associated with the most trustworthy effects.

### Content components of nurse counseling

Most frequently, counseling addressed treatment-related physical symptoms [25, 42–48], symptom etiology [25, 26, 41, 43–47], symptom prevention and/or treatment [25, 26, 41–48], and utilization of healthcare services [25, 26, 42–46]. Nurses and patients may see these themes as being at the core of nursing practice and competence. Moreover, many interventions were carried out around the time of active treatment [25, 26, 41–45, 47, 48], during which questions, such as what physical symptoms should be expected, and how they develop, how they are preventable and treatable, may have been more prominent than other themes. All these frequently used components were associated with significant effects in the studies providing the best available evidence [25, 43, 44].

In few interventions, counseling included disease etiology [42, 46], diagnostic or treatment-related procedures [26, 42, 46], treatment-related decision-making [43, 44], as well as goal setting and planning within symptom management [25, 43, 44]. Interestingly these themes were only discussed in interventions with academically educated nurses. We assume that disease and treatment-related issues (e.g., diagnostics) were not addressed in other interventions because they are traditionally more assigned to physicians’ competence. In particular, health-related decision-making can be an important theme when patients are in a dilemma to help them determine which options represent the most satisfying outcome for them [58]. However, in the most trustworthy studies, symptom-management was addressed comprehensively [25, 43, 44], whereas both the inclusion [43, 44] and exclusion [25] of health-related decision-making was associated with positive effects.

### Limitations

The results of this systematic review are limited to women with gynecologic cancer and our restrictive definition of pre-cancer excluded studies concerning less rigorously confirmed conditions. On the one hand, it was critical to draw the line at colposcopy/biopsy-confirmed conditions, and on the other hand, it was not reasonable to exclude women with pre-cancer from the research interest of this review since they need to be considered in gynecologic oncology. Furthermore, a broad concept of counseling was adopted. Thus, included interventions can also be characterized as psychosocial or supportive programs. Additionally, pre-experimental studies were eligible, even though these generally exhibit less internal validity than experimental investigations. The additional objective of identifying counseling components justified the sourcing of all available intervention studies using quantitative designs. Moreover, it was not feasible to reconstruct which effects on symptoms were observed in quality of life measures since studies did not report findings of single items. Partially limited study reporting also restricted the data synthesis regarding the structure and content of nurse counseling.

## Conclusions

The current evidence base about the effectiveness of nurse counseling appears to be too fragmented and inconsistent to establish comprehensive implications for practice. Nevertheless, the results suggest that nurse counseling ameliorates symptom distress. For women exhibiting depressive symptoms, referral to appropriate psychological services is recommended. Patient outcomes may be improved by nurse counselors with an academic education administering repeated consultations in an individual format during and after active treatment by including structured, tailored, interdisciplinary orientated, and theoretically based counseling concepts, as well as specific materials, a comprehensive management of disease and treatment-related symptoms and the utilization of healthcare services. More well-designed, large-scale studies including women with confirmed pre-cancer, based on a theoretical model of the intervention effect thereafter translated in the selection of outcome measures, are required. Moreover, combined and innovative counseling forms involving the social environment, various tools and materials, issues touching on physicians’ competence, and specific symptom management strategies remain an issue for future studies. In particular, the identified components can be used in future studies as well as by healthcare providers for the systematic development of nurse counseling as a complex intervention. Therefore, it must be carefully determined how to combine and adopt various components for specific patient groups.

## Additional files

Additional file 1: Critical appraisal tools. Description of data: Standardized Critical Appraisal Checklist from the JBI. (DOCX 18 kb)

Additional file 2: Levels of evidence for effectiveness. Description of data: JBI levels of evidence. (DOCX 14 kb)

Additional file 3: Critical appraisal of included studies. Description of data: Critical appraisal of included experimental and pre-experimental studies. (DOCX 43 kb)

Additional file 4: Outcomes listed by intervention effects. Description of data: A table listing all identified outcomes by significant and non-significant improvements due to the tested interventions.) (DOCX 67 kb)

Additional file 5: Structure and content components associated with significant and non-significant effects. Description of data: A table listing all components by their effects on all measured outcomes within included studies. (DOCX 565 kb)

## Abbreviations

APN: Advanced practice nurse
JBI: Joanna Briggs Institute
MAStARI: Meta analysis of statistics assessment and review instrument
MeSH: Medical subject heading
