# Supplementary material for: Effectiveness, structure, and content of nurse counseling in gynecologic oncology: a systematic review
Source: BMC Nurs. 2017 Aug 3;16:43. doi: 10.1186/s12912-017-0237-z (PMC5543445; doi:10.1186/s12912-017-0237-z)
Supplement: Supplementary file 1 — Critical appraisal tools. Description of data: Standardized Critical Appraisal Checklist from the JBI. (DOCX 18 kb) [file 12912_2017_237_MOESM1_ESM.docx]

**Additional file 1. Critical appraisal tools**

Standardized Critical Appraisal Checklist from the JBI - MAStARI (Joanna Briggs Institute - Meta Analysis of Statistics Assessment and Review Instrument) – **Randomized Control / Pseudo-randomized Trial**

Reviewer:

Date:

Study reference:

| **Criteria** | **Yes** | **No** | **Unclear** | **Not applicable** | **Comments** |
| --- | --- | --- | --- | --- | --- |
| 1. Was the assignment to treatment group truly random? | 🞏 | 🞏 | 🞏 | 🞏 |  |
| 1. Were participants blinded to treatment allocation? | 🞏 | 🞏 | 🞏 | 🞏 |  |
| 1. Was allocation to treatment groups concealed from the allocator? | 🞏 | 🞏 | 🞏 | 🞏 |  |
| 1. Were the outcomes of people who withdrew described and included in the analysis? | 🞏 | 🞏 | 🞏 | 🞏 |  |
| 1. Were those assessing outcome blind to the treatment allocation? | 🞏 | 🞏 | 🞏 | 🞏 |  |
| 1. Were the control and treatment groups comparable at entry? | 🞏 | 🞏 | 🞏 | 🞏 |  |
| 1. Were groups treated identically other than for the named interventions? | 🞏 | 🞏 | 🞏 | 🞏 |  |
| 1. Were outcomes measured in the same ways for all groups? | 🞏 | 🞏 | 🞏 | 🞏 |  |
| 1. Were outcomes measured in a reliable way? | 🞏 | 🞏 | 🞏 | 🞏 |  |
| 1. Was appropriate statistical analysis used? | 🞏 | 🞏 | 🞏 | 🞏 |  |

Overall appraisal: Included 🞏 Excluded 🞏 Seek further information 🞏

Standardized Critical Appraisal Checklist from the JBI- MAStARI (Joanna Briggs Institute - Meta Analysis of Statistics Assessment and Review Instrument) – **Descriptive / Case Series Studies**

Reviewer:

Date:

Study reference:

| **Criteria** | **Yes** | **No** | **Unclear** | **Not applicable** | **Comments** |
| --- | --- | --- | --- | --- | --- |
| 1. Was study based on random or pseudo-random sample? | 🞏 | 🞏 | 🞏 | 🞏 |  |
| 1. Were the criteria for inclusion in the sample clearly defined? | 🞏 | 🞏 | 🞏 | 🞏 |  |
| 1. Were confounding factors identified and strategies to deal with them stated? | 🞏 | 🞏 | 🞏 | 🞏 |  |
| 1. Were outcomes assessed using objective criteria? | 🞏 | 🞏 | 🞏 | 🞏 |  |
| 1. If comparisons are being made, was there sufficient descriptions of the groups? | 🞏 | 🞏 | 🞏 | 🞏 |  |
| 1. Was follow-up carried out over a sufficient time period? | 🞏 | 🞏 | 🞏 | 🞏 |  |
| 1. Were the outcomes of people who withdrew described and included in the analysis? | 🞏 | 🞏 | 🞏 | 🞏 |  |
| 1. Were outcomes measured in a reliable way? | 🞏 | 🞏 | 🞏 | 🞏 |  |
| 1. Was appropriate statistical analysis used? | 🞏 | 🞏 | 🞏 | 🞏 |  |

Overall appraisal: Included 🞏 Excluded 🞏 Seek further information 🞏
