# Supplementary material for: Effectiveness, structure, and content of nurse counseling in gynecologic oncology: a systematic review
Source: BMC Nurs. 2017 Aug 3;16:43. doi: 10.1186/s12912-017-0237-z (PMC5543445; doi:10.1186/s12912-017-0237-z)
Supplement: Supplementary file 2 — Levels of evidence for effectiveness. Description of data: JBI levels of evidence. (DOCX 14 kb) [file 12912_2017_237_MOESM2_ESM.docx]

**Additional file 2. Levels of evidence for effectiveness**

**JBI levels of evidence**

| **Level** | **Study design** |
| --- | --- |
| *Level 1* | *Experimental designs* |
| Level 1.a | Systematic review of randomized controlled trials (RCTs) |
| Level 1.b | Systematic review of RCTs and other study designs |
| Level 1.c | RCT |
| Level 1.d | Pseudo-RCT |
| *Level 2* | *Quasi-experimental Designs* |
| Level 2.a | Systematic review of quasi-experimental studies |
| Level 2.b | Systematic review of quasi-experimental and other study designs |
| Level 2.c | Quasi-experimental prospective controlled study |
| Level 2.d | Pre-test - post-test or historic//retrospective control group study |
| *Level 3* | *Observational – analytic designs* |
| Level 3.a | Systematic review of comparable cohort studies |
| Level 3.b | Systematic review of comparable cohort and other lower study designs |
| Level 3.c | Cohort study with control group |
| Level 3.d | Case – controlled study |
| *Level 4* | *Observational – descriptive studies* |
| Level 4.a | Systematic review of descriptive studies |
| Level 4.b | Cross-sectional study |
| Level 4.c | Case series |
| Level 4.d | Case study |
| *Level 5* | *Expert Opinion and Bench Research* |
| Level 5.a | Systematic review of expert opinion |
| Level 5.b | Expert consensus |
| Level 5.c | Bench research/single expert opinion |
