# Supplementary material for: Effectiveness, structure, and content of nurse counseling in gynecologic oncology: a systematic review
Source: BMC Nurs. 2017 Aug 3;16:43. doi: 10.1186/s12912-017-0237-z (PMC5543445; doi:10.1186/s12912-017-0237-z)
Supplement: Supplementary file 3 — Critical appraisal of included studies. Description of data: Critical appraisal of included experimental and pre-experimental studies. (DOCX 43 kb) [file 12912_2017_237_MOESM3_ESM.docx]

**Additional file 3. Critical appraisal of included studies**

**Critical appraisal of included experimental studies**

| **Criteria** | Aktaş et al. 2014 [41] | Chow et al. 2014 [42] | Donovan et al. 2014 [25] | Maughan et al. 2001 [26] | McCorkle et al. 2009 [43] | McCorkle et al. 2011 [44] | Nolte et al. 2006 [45] |
| --- | --- | --- | --- | --- | --- | --- | --- |
| Randomization | U^a^/U^b^ | Y^a^/Y^b^ | Y^a^/Y^b^ | Y^a^/Y^b^ | Y^a^/Y^b^ | Y^a^/Y^b^ | Y^a^/Y^b^ |
| Blinding of participants | U^a^/U^b^ | N^a^/N^b^ | U^a^/U^b^/N^d^ | U^a^/U^b^/N^d^ | Y^a^/N^b^/Y^c^ | Y^a^/N^b^/Y^c^ | U^a^/U^b^/N^d^ |
| Allocation concealment | U^a^/U^b^ | Y^a^/Y^b^ | Y^a^/Y^b^ | Y^a^/Y^b^ | Y^a^/Y^b^ | Y^a^/Y^b^ | Y^a^/Y^b^ |
| Intention to treat analysis | N^a^/U^b^/N^c^ | Y^a^/Y^b^ | Y^a^/Y^b^ | N^a^/U^b^/N^c^ | U^a^/U^b^/Y^d^ | U^a^/U^b^/Y^d^ | U^a^/U^b^/Y^d^ |
| Blinding of outcome assessor | U^a^/U^b^ | N^a^/N^b^ | Y^a^/Y^b^ | U^a^/U^b^/Y^d^ | Y^a^/Y^b^ | Y^a^/Y^b^ | U^a^/U^b^/N^d^ |
| Similarity at baseline | Y^a^/Y^b^ | N^a^/Y^b^/Y^c^ | Y^a^/Y^b^ | N^a^/N^b^ | N^a^/N^b^ | N^a^/N^b^ | U^a^/U^b^/Y^d^ |
| Groups treated identically other than intervention | Y^a^/Y^b^ | U^a^/U^b^/Y^d^ | Y^a^/Y^b^ | Y^a^/Y^b^ | Y^a^/Y^b^ | Y^a^/Y^b^ | Y^a^/Y^b^ |
| Outcomes measured same for all groups | U^a^/Y^a^/U^c^ | Y^a^/Y^b^ | Y^a^/Y^b^ | Y^a^/Y^b^ | Y^a^/Y^b^ | Y^a^/Y^b^ | U^a^/U^b^/Y^d^ |
| Outcomes measured reliable | Y^a^/Y^b^ | U^a^/U^b^ | Y^a^/Y^b^ | U^a^/N^b^/U^c^ | Y^a^/Y^b^ | Y^a^/Y^b^ | U^a^/U^b^ |
| Appropriate statistical analysis | Y^a^/Y^b^ | Y^a^/Y^b^ | Y^a^/Y^b^ | Y^a^/Y^b^ | Y^a^/Y^b^ | Y^a^/Y^b^ | Y^a^/Y^b^ |
| Cohens's kappa^e^ | 0.64 | 0.83 | 1.00 | 0.68 | 0.76 | 0.76 | 1.00 |
| Total quality score (*n*)^f^ | 4 | 7 | 9 | 6 | 9 | 9 | 7 |
| Total quality score (%)^g^ | 40 | 70 | 90 | 60 | 90 | 90 | 70 |
| Study quality^h^ | Low | Moderate | High | Moderate | High | High | Moderate |

N = No; Y = Yes; U = Unclear

^a^Reviewer 1.

^b^Reviewer 2.

^c^Consensus between reviewer 1 and 2.

^d^Information provided by study authors.

^e^Interrater-reliability between reviewer 1 and 2.

^f^Sum scores can range from 0 to 10; higher scores indicate higher methodological study quality.

^g^Sum scores transformed in percent values; Percentage scores can range from 0 to 100%; higher scores indicate higher methodological study quality.

^h^Low methodological quality refers to total quality scores ranging from 0 to 49%; Moderate methodological quality refers to total quality scores ranging from 50 to 79%; high methodological quality refers to total quality scores ranging from 80 to 100%.

**Critical appraisal of included pre-experimental studies**

| **Criteria** | Cox et al. 2008 [46] | Liu et al. 2001 [48] | So et al. 2006 [47] |
| --- | --- | --- | --- |
| Random or pseudo-random sample | NA^a^/NA^b^ | NA^a^/NA^b^ | NA^a^/NA^b^ |
| Inclusion criteria clearly stated | Y^a^/Y^b^ | Y^a^/Y^b^ | Y^a^/Y^b^ |
| Confounding factors identified and strategies to control stated | N^a^/U^b^/U^c^ | N^a^/N^b^ | Y^a^/Y^b^ |
| Outcomes assessed using objective criteria | Y^a^/Y^b^ | Y^a^/Y^b^ | Y^a^/Y^b^ |
| Sufficient description of groups | NA^a^/NA^b^ | Y^a^/Y^b^ | NA^a^/NA^b^ |
| Follow-up over a sufficient time period | Y^a^/Y^b^ | Y^a^/Y^b^ | U^a^/U^b^ |
| Intention to treat analysis | N^a^/N^b^ | N^a^/N^b^ | N^a^/U^b^/U^c^ |
| Outcomes measured reliable | U^a^/U^b^ | U^a^/U^b^ | U^a^/U^b^ |
| Appropriate statistical analysis | U^a^/U^b^ | Y^a^/Y^b^ | Y^a^/Y^b^ |
| Cohens's kappa^e^ | 0.85 | 1.00 | 0.84 |
| Total quality score (*n*)^f^ | 3 | 5 | 4 |
| Total quality score (%)^g^ | 42.9 | 62.5 | 57.1 |
| Study quality^h^ | Low | Moderate | Moderate |

N = No; Y = Yes; U = Unclear; NA = Not Applicable

^a^Reviewer 1.

^b^Reviewer 2.

^c^Consensus between reviewer 1 and 2.

^d^Information provided by study authors.

^e^Interrater-reliability between reviewer 1 and 2.

^f^Sum scores can range from 0 to 10; higher scores indicate higher methodological study quality.

^g^Sum scores transformed in percent values; Percentage scores can range from 0 to 100%; higher scores indicate higher methodological study quality.

^h^Low methodological quality = total quality scores ranging from 0 to 49%; Moderate methodological quality = total quality scores ranging from 50 to 79%; high methodological quality = total quality scores ranging from 80 to 100%.
