# Supplementary material for: Effectiveness, structure, and content of nurse counseling in gynecologic oncology: a systematic review
Source: BMC Nurs. 2017 Aug 3;16:43. doi: 10.1186/s12912-017-0237-z (PMC5543445; doi:10.1186/s12912-017-0237-z)
Supplement: Supplementary file 4 — Outcomes listed by intervention effects. Description of data: A table listing all identified outcomes by significant and non-significant improvements due to the tested interventions.) (DOCX 67 kb) [file 12912_2017_237_MOESM4_ESM.docx]

**Additional file 4: Outcomes listed by intervention effects**

| **Outcome** | **Comparison** | **Effect** | **Evidence level^a^/Study quality^b^** | **Reference** |
| --- | --- | --- | --- | --- |
| Quality of life – overall | Attention control | No significant improvement | 1c/high | [43] |
| Quality of life – overall | Standard care | Significant improvement | 1c/moderate | [26] |
| Quality of life – overall | Attention control | No significant improvement | 1c/moderate | [42] |
| Quality of life – overall | Pretest/posttest | No significant improvement | 4c/low | [46] |
| Quality of life – physical | Attention control | Significant improvement | 1c/high | [43] |
| Quality of life – physical | Standard care | No significant improvement | 1c/moderate | [26] |
| Quality of life – physical | Attention control | No significant improvement | 1c/moderate | [42] |
| Quality of life – physical | Pretest/posttest | No significant improvement | 4c/low | [46] |
| Quality of life – functional | Attention control | No significant improvement | 1c/moderate | [42] |
| Quality of life – functional | Pretest/posttest | No significant improvement | 4c/low | [46] |
| Quality of life - cognitive | Attention control | Significant improvement | 1c/high | [43] |
| Quality of life - cognitive | Standard care | No significant improvement | 1c/moderate | [26] |
| Quality of life – emotional | Attention control | No significant improvement | 1c/moderate | [42] |
| Quality of life – emotional | Standard care | No significant improvement | 1c/moderate | [26] |
| Quality of life – emotional | Pretest/posttest | Significant improvement | 4c/low | [46] |
| Quality of life – social | Attention control | No significant improvement | 1c/moderate | [42] |
| Quality of life – social | Standard care | No significant improvement | 1c/moderate | [26] |
| Quality of life – social | Pretest/posttest | No significant improvement | 4c/low | [46] |
| Quality of life – sexual | Standard care | No significant improvement | 1c/moderate | [26] |
| Quality of life - symptoms | Pretest/posttest | No significant improvement | 4c/low | [46] |
| Urinalysis | Intervention II | Significant improvement | 4b/moderate | [48] |
| Urine culture | Intervention II | No significant improvement | 4b/moderate | [48] |
| Uncertainty – overall | Attention control | No significant improvement | 1c/moderate | [42] |
| Uncertainty – ambiguity | Attention control | Significant improvement | 1c/high | [43] |
| Uncertainty – ambiguity | Attention control | No significant improvement | 1c/moderate | [42] |
| Uncertainty – complexity | Attention control | No significant improvement | 1c/moderate | [42] |
| Uncertainty – unpredictability | Attention control | No significant improvement | 1c/moderate | [42] |
| Uncertainty - inconsistency | Attention control | Significant improvement | 1c/moderate | [42] |
| Depression | Attention control | No significant improvement | 1c/high | [43] |
| Depression | Attention control | No significant improvement | 1c/moderate | [42] |
| Body image | Intervention II | No significant improvement | 1c/moderate | [45] |
| Self-esteem | Intervention II | No significant improvement | 1c/moderate | [45] |
| Sexual functioning | Attention control | No significant improvement | 1c/moderate | [42] |
| Sexual functioning | Standard care | No significant improvement | 1c/moderate | [26] |
| Sexual satisfaction | Standard care | Significant improvement | 1c/low | [41] |
| Knowledge | Intervention II | Significant improvement | 4b/moderate | [48] |
| Knowledge | Pretest/posttest | Significant improvement | 4c/moderate | [47] |
| Attitude | Pretest/posttest | Significant improvement | 4c/moderate | [47] |
| Social support | Attention control | No significant improvement | 1c/moderate | [42] |
| Symptom severity | Standard care | Significant improvement | 1c/high | [25] |
| Symptom distress | Standard care | Significant improvement | 1c/high | [25] |
| Symptom distress | Attention control | Significant improvement | 1c/high | [43] |
| Symptom consequences | Standard care | No significant improvement | 1c/high | [25] |
| Symptom controllability | Standard care | No significant improvement | 1c/high | [25] |
| Self-care performance | Intervention II | No significant improvement | 4b/moderate | [48] |
| Hospitalizations | Attention control | No significant improvement | 1c/high | [44] |
| Oncology outpatient visits | Attention control | No significant improvement | 1c/high | [44] |
| Emergency room visits | Attention control | No significant improvement | 1c/high | [44] |
| Primary care visits | Attention control | Significant improvement | 1c/high | [44] |

**^a^**Level 1 refers to experimental designs, level 2 to quasi-experimental designs, level 3 to observational-analytic designs, level 4 to observational-descriptive studies, and level 5 to expert opinion and bench research [33].

^b^Low methodological quality refers to total quality scores ranging from 0 to 49%; Moderate methodological quality refers to total quality scores ranging from 50 to 79%; high methodological quality refers to total quality scores ranging from 80 to 100%.
