# Supplementary material for: Effectiveness, structure, and content of nurse counseling in gynecologic oncology: a systematic review
Source: BMC Nurs. 2017 Aug 3;16:43. doi: 10.1186/s12912-017-0237-z (PMC5543445; doi:10.1186/s12912-017-0237-z)
Supplement: Supplementary file 5 — Structure and content components associated with significant and non-significant effects. Description of data: A table listing all components by their effects on all measured outcomes within included studies. (DOCX 565 kb) [file 12912_2017_237_MOESM5_ESM.docx]

**Additional file 5. Structure and content components associated with significant and non-significant effects**

| **Structure components** | **Outcome** | **Comparison** | **Effect** | **Study quality score** | **Study quality** | **Evidence level** | **Risk type II errors** | **Reference** |
| --- | --- | --- | --- | --- | --- | --- | --- | --- |
| **Provider** |  |  |  |  |  |  |  |  |
| Non-specialized nurse without academic education | Knowledge | Intervention I/intervention II | Significant | 5 | Moderate | 4b | No | [48] |
|  | Self-care performance | Intervention I/intervention II | Non-significant | 5 | Moderate | 4b | Yes | [48] |
|  | Urinalysis | Intervention I/intervention II | Non-significant | 5 | Moderate | 4b | Yes | [48] |
|  | Urine culture | Intervention I/intervention II | Non-significant | 5 | Moderate | 4b | Yes | [48] |
| Non-specialized nurse with academic education | Symptom severity | Standard care | Significant | 9 | High | 1c | No | [25] |
|  | Symptom distress | Standard care | Significant | 9 | High | 1c | No | [25] |
|  | Symptom consequences | Standard care | Non-significant | 9 | High | 1c | No | [25] |
|  | Symptom controllability | Standard care | Non-significant | 9 | High | 1c | No | [25] |
|  | Sexual functioning | Attention control | Non-significant | 7 | High | 1c | Yes | [42] |
|  | Uncertainty | Attention control | Partly significant | 7 | High | 1c | Yes | [42] |
|  | Anxiety/depression | Attention control | Non-significant | 7 | High | 1c | Yes | [42] |
|  | Social support | Attention control | Non-significant | 7 | High | 1c | Yes | [42] |
|  | Overall quality of life | Attention control | Non-significant | 7 | High | 1c | Yes | [42] |
|  | Physical quality of life | Attention control | Non-significant | 7 | High | 1c | Yes | [42] |
|  | Functional quality of life | Attention control | Non-significant | 7 | High | 1c | Yes | [42] |
|  | Emotional quality of life | Attention control | Non-significant | 7 | High | 1c | Yes | [42] |
|  | Social quality of life | Attention control | Non-significant | 7 | High | 1c | Yes | [42] |
| Specialized nurse with academic education | Overall quality of life | Standard care | Significant | 6 | Moderate | 1c | No | [26] |
|  | Physical quality of life | Standard care | Non-significant | 6 | Moderate | 1c | Yes | [26] |
|  | Cognitive quality of life | Standard care | Non-significant | 6 | Moderate | 1c | Yes | [26] |
|  | Emotional quality of life | Standard care | Non-significant | 6 | Moderate | 1c | Yes | [26] |
|  | Social quality of life | Standard care | Non-significant | 6 | Moderate | 1c | Yes | [26] |
|  | Sexual quality of life | Standard care | Non-significant | 6 | Moderate | 1c | Yes | [26] |
|  | Sexual functioning | Standard care | Non-significant | 6 | Moderate | 1c | Yes | [26] |
|  | Depression | Attention control | Non-significant | 9 | High | 1c | No | [43] |
|  | Uncertainty - ambiguity | Attention control | Significant | 9 | High | 1c | No | [43] |
|  | Symptom distress | Attention control | Significant | 9 | High | 1c | No | [43] |
|  | Overall quality of life | Attention control | Non-significant | 9 | High | 1c | No | [43] |
|  | Physical quality of life | Attention control | Significant | 9 | High | 1c | No | [43] |
|  | Cognitive quality of life | Attention control | Significant | 9 | High | 1c | No | [43] |
|  | Hospitalizations | Attention control | Non-significant | 9 | High | 1c | No | [44] |
|  | Oncology outpatient visits | Attention control | Non-significant | 9 | High | 1c | No | [44] |
|  | Emergency room visits | Attention control | Non-significant | 9 | High | 1c | No | [44] |
|  | Primary care visits | Attention control | Significant | 9 | High | 1c | No | [44] |
|  | Overall quality of life | Pretest/posttest | Non-significant | 3 | Low | 4c | No | [46] |
|  | Physical quality of life | Pretest/posttest | Non-significant | 3 | Low | 4c | No | [46] |
|  | Functional quality of life | Pretest/posttest | Non-significant | 3 | Low | 4c | No | [46] |
|  | Emotional quality of life | Pretest/posttest | Significant | 3 | Low | 4c | No | [46] |
|  | Social quality of life | Pretest/posttest | Non-significant | 3 | Low | 4c | No | [46] |
|  | Quality of life - symptoms | Pretest/posttest | Non-significant | 3 | Low | 4c | No | [46] |
| **Receiver** |  |  |  |  |  |  |  |  |
| Patients only | Symptom severity | Standard care | Significant | 9 | High | 1c | No | [25] |
|  | Symptom distress | Standard care | Significant | 9 | High | 1c | No | [25] |
|  | Symptom consequences | Standard care | Non-significant | 9 | High | 1c | No | [25] |
|  | Symptom controllability | Standard care | Non-significant | 9 | High | 1c | No | [25] |
|  | Sexual satisfaction | Standard care | Significant | 4 | Low | 1c | No | [41] |
|  | Sexual functioning | Attention control | Non-significant | 7 | High | 1c | Yes | [42] |
|  | Uncertainty | Attention control | Partly significant | 7 | High | 1c | Yes | [42] |
|  | Anxiety/depression | Attention control | Non-significant | 7 | High | 1c | Yes | [42] |
|  | Social support | Attention control | Non-significant | 7 | High | 1c | Yes | [42] |
|  | Overall quality of life | Attention control | Non-significant | 7 | High | 1c | Yes | [42] |
|  | Physical quality of life | Attention control | Non-significant | 7 | High | 1c | Yes | [42] |
|  | Functional quality of life | Attention control | Non-significant | 7 | High | 1c | Yes | [42] |
|  | Emotional quality of life | Attention control | Non-significant | 7 | High | 1c | Yes | [42] |
|  | Social quality of life | Attention control | Non-significant | 7 | High | 1c | Yes | [42] |
|  | Body image | Intervention I/intervention II | Non-significant | 7 | Moderate | 1c | No | [45] |
|  | Self-esteem | Intervention I/intervention II | Non-significant | 7 | Moderate | 1c | No | [45] |
|  | Overall quality of life | Pretest/posttest | Non-significant | 3 | Low | 4c | No | [46] |
|  | Physical quality of life | Pretest/posttest | Non-significant | 3 | Low | 4c | No | [46] |
|  | Functional quality of life | Pretest/posttest | Non-significant | 3 | Low | 4c | No | [46] |
|  | Emotional quality of life | Pretest/posttest | Significant | 3 | Low | 4c | No | [46] |
|  | Social quality of life | Pretest/posttest | Non-significant | 3 | Low | 4c | No | [46] |
|  | Quality of life - symptoms | Pretest/posttest | Non-significant | 3 | Low | 4c | No | [46] |
|  | Knowledge | Intervention I/intervention II | Significant | 5 | Moderate | 4b | No | [48] |
|  | Self-care performance | Intervention I/intervention II | Non-significant | 5 | Moderate | 4b | Yes | [48] |
|  | Urinalysis | Intervention I/intervention II | Non-significant | 5 | Moderate | 4b | Yes | [48] |
|  | Urine culture | Intervention I/intervention II | Non-significant | 5 | Moderate | 4b | Yes | [48] |
| Patients and families | Overall quality of life | Standard care | Significant | 6 | Moderate | 1c | No | [26] |
|  | Physical quality of life | Standard care | Non-significant | 6 | Moderate | 1c | Yes | [26] |
|  | Cognitive quality of life | Standard care | Non-significant | 6 | Moderate | 1c | Yes | [26] |
|  | Emotional quality of life | Standard care | Non-significant | 6 | Moderate | 1c | Yes | [26] |
|  | Social quality of life | Standard care | Non-significant | 6 | Moderate | 1c | Yes | [26] |
|  | Sexual quality of life | Standard care | Non-significant | 6 | Moderate | 1c | Yes | [26] |
|  | Sexual functioning | Standard care | Non-significant | 6 | Moderate | 1c | Yes | [26] |
|  | Depression | Attention control | Non-significant | 9 | High | 1c | No | [43] |
|  | Uncertainty - ambiguity | Attention control | Significant | 9 | High | 1c | No | [43] |
|  | Symptom distress | Attention control | Significant | 9 | High | 1c | No | [43] |
|  | Overall quality of life | Attention control | Non-significant | 9 | High | 1c | No | [43] |
|  | Physical quality of life | Attention control | Significant | 9 | High | 1c | No | [43] |
|  | Cognitive quality of life | Attention control | Significant | 9 | High | 1c | No | [43] |
|  | Hospitalizations | Attention control | Non-significant | 9 | High | 1c | No | [44] |
|  | Oncology outpatient visits | Attention control | Non-significant | 9 | High | 1c | No | [44] |
|  | Emergency room visits | Attention control | Non-significant | 9 | High | 1c | No | [44] |
|  | Primary care visits | Attention control | Significant | 9 | High | 1c | No | [44] |
|  | Knowledge | Pretest/posttest | Significant | 4 | Moderate | 4c | No | [47] |
|  | Attitude | Pretest/posttest | Significant | 4 | Moderate | 4c | No | [47] |
| **Time frame** |  |  |  |  |  |  |  |  |
| Before active treatment only | Body image | Intervention I/intervention II | Non-significant | 7 | Moderate | 1c | No | [45] |
|  | Self-esteem | Intervention I/intervention II | Non-significant | 7 | Moderate | 1c | No | [45] |
|  | Knowledge | Pretest/posttest | Significant | 4 | Moderate | 4c | No | [47] |
|  | Attitude | Pretest/posttest | Significant | 4 | Moderate | 4c | No | [47] |
| During active treatment only | Knowledge | Intervention I/intervention II | Significant | 5 | Moderate | 4b | No | [48] |
|  | Self-care performance | Intervention I/intervention II | Non-significant | 5 | Moderate | 4b | Yes | [48] |
|  | Urinalysis | Intervention I/intervention II | Non-significant | 5 | Moderate | 4b | Yes | [48] |
|  | Urine culture | Intervention I/intervention II | Non-significant | 5 | Moderate | 4b | Yes | [48] |
| After active treatment only | Overall quality of life | Pretest/posttest | Non-significant | 3 | Low | 4c | No | [46] |
|  | Physical quality of life | Pretest/posttest | Non-significant | 3 | Low | 4c | No | [46] |
|  | Functional quality of life | Pretest/posttest | Non-significant | 3 | Low | 4c | No | [46] |
|  | Emotional quality of life | Pretest/posttest | Significant | 3 | Low | 4c | No | [46] |
|  | Social quality of life | Pretest/posttest | Non-significant | 3 | Low | 4c | No | [46] |
|  | Quality of life - symptoms | Pretest/posttest | Non-significant | 3 | Low | 4c | No | [46] |
| During and after active treatment | Depression | Attention control | Non-significant | 9 | High | 1c | No | [43] |
|  | Uncertainty - ambiguity | Attention control | Significant | 9 | High | 1c | No | [43] |
|  | Symptom distress | Attention control | Significant | 9 | High | 1c | No | [43] |
|  | Overall quality of life | Attention control | Non-significant | 9 | High | 1c | No | [43] |
|  | Physical quality of life | Attention control | Significant | 9 | High | 1c | No | [43] |
|  | Cognitive quality of life | Attention control | Significant | 9 | High | 1c | No | [43] |
|  | Hospitalizations | Attention control | Non-significant | 9 | High | 1c | No | [44] |
|  | Oncology outpatient visits | Attention control | Non-significant | 9 | High | 1c | No | [44] |
|  | Emergency room visits | Attention control | Non-significant | 9 | High | 1c | No | [44] |
|  | Primary care visits | Attention control | Significant | 9 | High | 1c | No | [44] |
| Before, during, and after active treatment | Symptom severity | Standard care | Significant | 9 | High | 1c | No | [25] |
|  | Symptom distress | Standard care | Significant | 9 | High | 1c | No | [25] |
|  | Symptom consequences | Standard care | Non-significant | 9 | High | 1c | No | [25] |
|  | Symptom controllability | Standard care | Non-significant | 9 | High | 1c | No | [25] |
|  | Overall quality of life | Standard care | Significant | 6 | Moderate | 1c | No | [26] |
|  | Physical quality of life | Standard care | Non-significant | 6 | Moderate | 1c | Yes | [26] |
|  | Cognitive quality of life | Standard care | Non-significant | 6 | Moderate | 1c | Yes | [26] |
|  | Emotional quality of life | Standard care | Non-significant | 6 | Moderate | 1c | Yes | [26] |
|  | Social quality of life | Standard care | Non-significant | 6 | Moderate | 1c | Yes | [26] |
|  | Sexual quality of life | Standard care | Non-significant | 6 | Moderate | 1c | Yes | [26] |
|  | Sexual functioning | Standard care | Non-significant | 6 | Moderate | 1c | Yes | [26] |
|  | Sexual satisfaction | Standard care | Significant | 4 | Low | 1c | No | [41] |
|  | Sexual functioning | Attention control | Non-significant | 7 | High | 1c | Yes | [42] |
|  | Uncertainty | Attention control | Partly significant | 7 | High | 1c | Yes | [42] |
|  | Anxiety/depression | Attention control | Non-significant | 7 | High | 1c | Yes | [42] |
|  | Social support | Attention control | Non-significant | 7 | High | 1c | Yes | [42] |
|  | Overall quality of life | Attention control | Non-significant | 7 | High | 1c | Yes | [42] |
|  | Physical quality of life | Attention control | Non-significant | 7 | High | 1c | Yes | [42] |
|  | Functional quality of life | Attention control | Non-significant | 7 | High | 1c | Yes | [42] |
|  | Emotional quality of life | Attention control | Non-significant | 7 | High | 1c | Yes | [42] |
|  | Social quality of life | Attention control | Non-significant | 7 | High | 1c | Yes | [42] |
| One-time consultation | Body image | Intervention I/intervention II | Non-significant | 7 | Moderate | 1c | No | [45] |
|  | Self-esteem | Intervention I/intervention II | Non-significant | 7 | Moderate | 1c | No | [45] |
|  | Knowledge | Pretest/posttest | Significant | 4 | Moderate | 4c | No | [47] |
|  | Attitude | Pretest/posttest | Significant | 4 | Moderate | 4c | No | [47] |
| Repeated consultation | Symptom severity | Standard care | Significant | 9 | High | 1c | No | [25] |
|  | Symptom distress | Standard care | Significant | 9 | High | 1c | No | [25] |
|  | Symptom consequences | Standard care | Non-significant | 9 | High | 1c | No | [25] |
|  | Symptom controllability | Standard care | Non-significant | 9 | High | 1c | No | [25] |
|  | Overall quality of life | Standard care | Significant | 6 | Moderate | 1c | No | [26] |
|  | Physical quality of life | Standard care | Non-significant | 6 | Moderate | 1c | Yes | [26] |
|  | Cognitive quality of life | Standard care | Non-significant | 6 | Moderate | 1c | Yes | [26] |
|  | Emotional quality of life | Standard care | Non-significant | 6 | Moderate | 1c | Yes | [26] |
|  | Social quality of life | Standard care | Non-significant | 6 | Moderate | 1c | Yes | [26] |
|  | Sexual quality of life | Standard care | Non-significant | 6 | Moderate | 1c | Yes | [26] |
|  | Sexual functioning | Standard care | Non-significant | 6 | Moderate | 1c | Yes | [26] |
|  | Sexual satisfaction | Standard care | Significant | 4 | Low | 1c | No | [41] |
|  | Sexual functioning | Attention control | Non-significant | 7 | High | 1c | Yes | [42] |
|  | Uncertainty | Attention control | Partly significant | 7 | High | 1c | Yes | [42] |
|  | Anxiety/depression | Attention control | Non-significant | 7 | High | 1c | Yes | [42] |
|  | Social support | Attention control | Non-significant | 7 | High | 1c | Yes | [42] |
|  | Overall quality of life | Attention control | Non-significant | 7 | High | 1c | Yes | [42] |
|  | Physical quality of life | Attention control | Non-significant | 7 | High | 1c | Yes | [42] |
|  | Functional quality of life | Attention control | Non-significant | 7 | High | 1c | Yes | [42] |
|  | Emotional quality of life | Attention control | Non-significant | 7 | High | 1c | Yes | [42] |
|  | Social quality of life | Attention control | Non-significant | 7 | High | 1c | Yes | [42] |
|  | Depression | Attention control | Non-significant | 9 | High | 1c | No | [43] |
|  | Uncertainty - ambiguity | Attention control | Significant | 9 | High | 1c | No | [43] |
|  | Symptom distress | Attention control | Significant | 9 | High | 1c | No | [43] |
|  | Overall quality of life | Attention control | Non-significant | 9 | High | 1c | No | [43] |
|  | Physical quality of life | Attention control | Significant | 9 | High | 1c | No | [43] |
|  | Cognitive quality of life | Attention control | Significant | 9 | High | 1c | No | [43] |
|  | Hospitalizations | Attention control | Non-significant | 9 | High | 1c | No | [44] |
|  | Oncology outpatient visits | Attention control | Non-significant | 9 | High | 1c | No | [44] |
|  | Emergency room visits | Attention control | Non-significant | 9 | High | 1c | No | [44] |
|  | Primary care visits | Attention control | Significant | 9 | High | 1c | No | [44] |
|  | Overall quality of life | Pretest/posttest | Non-significant | 3 | Low | 4c | No | [46] |
|  | Physical quality of life | Pretest/posttest | Non-significant | 3 | Low | 4c | No | [46] |
|  | Functional quality of life | Pretest/posttest | Non-significant | 3 | Low | 4c | No | [46] |
|  | Emotional quality of life | Pretest/posttest | Significant | 3 | Low | 4c | No | [46] |
|  | Social quality of life | Pretest/posttest | Non-significant | 3 | Low | 4c | No | [46] |
|  | Quality of life - symptoms | Pretest/posttest | Non-significant | 3 | Low | 4c | No | [46] |
|  | Knowledge | Intervention I/intervention II | Significant | 5 | Moderate | 4b | No | [48] |
|  | Self-care performance | Intervention I/intervention II | Non-significant | 5 | Moderate | 4b | Yes | [48] |
|  | Urinalysis | Intervention I/intervention II | Non-significant | 5 | Moderate | 4b | Yes | [48] |
|  | Urine culture | Intervention I/intervention II | Non-significant | 5 | Moderate | 4b | Yes | [48] |
| Short consultation | Symptom severity | Standard care | Significant | 9 | High | 1c | No | [25] |
|  | Symptom distress | Standard care | Significant | 9 | High | 1c | No | [25] |
|  | Symptom consequences | Standard care | Non-significant | 9 | High | 1c | No | [25] |
|  | Symptom controllability | Standard care | Non-significant | 9 | High | 1c | No | [25] |
|  | Overall quality of life | Pretest/posttest | Non-significant | 3 | Low | 4c | No | [46] |
|  | Physical quality of life | Pretest/posttest | Non-significant | 3 | Low | 4c | No | [46] |
|  | Functional quality of life | Pretest/posttest | Non-significant | 3 | Low | 4c | No | [46] |
|  | Emotional quality of life | Pretest/posttest | Significant | 3 | Low | 4c | No | [46] |
|  | Social quality of life | Pretest/posttest | Non-significant | 3 | Low | 4c | No | [46] |
|  | Quality of life - symptoms | Pretest/posttest | Non-significant | 3 | Low | 4c | No | [46] |
| Long consultation | Sexual functioning | Attention control | Non-significant | 7 | High | 1c | Yes | [42] |
|  | Uncertainty | Attention control | Partly significant | 7 | High | 1c | Yes | [42] |
|  | Anxiety/depression | Attention control | Non-significant | 7 | High | 1c | Yes | [42] |
|  | Social support | Attention control | Non-significant | 7 | High | 1c | Yes | [42] |
|  | Overall quality of life | Attention control | Non-significant | 7 | High | 1c | Yes | [42] |
|  | Physical quality of life | Attention control | Non-significant | 7 | High | 1c | Yes | [42] |
|  | Functional quality of life | Attention control | Non-significant | 7 | High | 1c | Yes | [42] |
|  | Emotional quality of life | Attention control | Non-significant | 7 | High | 1c | Yes | [42] |
|  | Social quality of life | Attention control | Non-significant | 7 | High | 1c | Yes | [42] |
|  | Knowledge | Pretest/posttest | Significant | 4 | Moderate | 4c | No | [47] |
|  | Attitude | Pretest/posttest | Significant | 4 | Moderate | 4c | No | [47] |
| **Mode of delivery** |  |  |  |  |  |  |  |  |
| Face-to-face only | Overall quality of life | Standard care | Significant | 6 | Moderate | 1c | No | [26] |
|  | Physical quality of life | Standard care | Non-significant | 6 | Moderate | 1c | Yes | [26] |
|  | Cognitive quality of life | Standard care | Non-significant | 6 | Moderate | 1c | Yes | [26] |
|  | Emotional quality of life | Standard care | Non-significant | 6 | Moderate | 1c | Yes | [26] |
|  | Social quality of life | Standard care | Non-significant | 6 | Moderate | 1c | Yes | [26] |
|  | Sexual quality of life | Standard care | Non-significant | 6 | Moderate | 1c | Yes | [26] |
|  | Sexual functioning | Standard care | Non-significant | 6 | Moderate | 1c | Yes | [26] |
|  | Sexual satisfaction | Standard care | Significant | 4 | Low | 1c | No | [41] |
|  | Body image | Intervention I/intervention II | Non-significant | 7 | Moderate | 1c | No | [45] |
|  | Self-esteem | Intervention I/intervention II | Non-significant | 7 | Moderate | 1c | No | [45] |
|  | Knowledge | Pretest/posttest | Significant | 4 | Moderate | 4c | No | [47] |
|  | Attitude | Pretest/posttest | Significant | 4 | Moderate | 4c | No | [47] |
|  | Knowledge | Intervention I/intervention II | Significant | 5 | Moderate | 4b | No | [48] |
|  | Self-care performance | Intervention I/intervention II | Non-significant | 5 | Moderate | 4b | Yes | [48] |
|  | Urinalysis | Intervention I/intervention II | Non-significant | 5 | Moderate | 4b | Yes | [48] |
|  | Urine culture | Intervention I/intervention II | Non-significant | 5 | Moderate | 4b | Yes | [48] |
| Phone only | Overall quality of life | Pretest/posttest | Non-significant | 3 | Low | 4c | No | [46] |
|  | Physical quality of life | Pretest/posttest | Non-significant | 3 | Low | 4c | No | [46] |
|  | Functional quality of life | Pretest/posttest | Non-significant | 3 | Low | 4c | No | [46] |
|  | Emotional quality of life | Pretest/posttest | Significant | 3 | Low | 4c | No | [46] |
|  | Social quality of life | Pretest/posttest | Non-significant | 3 | Low | 4c | No | [46] |
|  | Quality of life - symptoms | Pretest/posttest | Non-significant | 3 | Low | 4c | No | [46] |
| Internet only | Symptom severity | Standard care | Significant | 9 | High | 1c | No | [25] |
|  | Symptom distress | Standard care | Significant | 9 | High | 1c | No | [25] |
|  | Symptom consequences | Standard care | Non-significant | 9 | High | 1c | No | [25] |
|  | Symptom controllability | Standard care | Non-significant | 9 | High | 1c | No | [25] |
| Face-to-face and phone | Sexual functioning | Attention control | Non-significant | 7 | High | 1c | Yes | [42] |
|  | Uncertainty | Attention control | Partly significant | 7 | High | 1c | Yes | [42] |
|  | Anxiety/depression | Attention control | Non-significant | 7 | High | 1c | Yes | [42] |
|  | Social support | Attention control | Non-significant | 7 | High | 1c | Yes | [42] |
|  | Overall quality of life | Attention control | Non-significant | 7 | High | 1c | Yes | [42] |
|  | Physical quality of life | Attention control | Non-significant | 7 | High | 1c | Yes | [42] |
|  | Functional quality of life | Attention control | Non-significant | 7 | High | 1c | Yes | [42] |
|  | Emotional quality of life | Attention control | Non-significant | 7 | High | 1c | Yes | [42] |
|  | Social quality of life | Attention control | Non-significant | 7 | High | 1c | Yes | [42] |
|  | Depression | Attention control | Non-significant | 9 | High | 1c | No | [43] |
|  | Uncertainty - ambiguity | Attention control | Significant | 9 | High | 1c | No | [43] |
|  | Symptom distress | Attention control | Significant | 9 | High | 1c | No | [43] |
|  | Overall quality of life | Attention control | Non-significant | 9 | High | 1c | No | [43] |
|  | Physical quality of life | Attention control | Significant | 9 | High | 1c | No | [43] |
|  | Cognitive quality of life | Attention control | Significant | 9 | High | 1c | No | [43] |
|  | Hospitalizations | Attention control | Non-significant | 9 | High | 1c | No | [44] |
|  | Oncology outpatient visits | Attention control | Non-significant | 9 | High | 1c | No | [44] |
|  | Emergency room visits | Attention control | Non-significant | 9 | High | 1c | No | [44] |
|  | Primary care visits | Attention control | Significant | 9 | High | 1c | No | [44] |
| **Format** |  |  |  |  |  |  |  |  |
| Individual counseling only | Symptom severity | Standard care | Significant | 9 | High | 1c | No | [25] |
|  | Symptom distress | Standard care | Significant | 9 | High | 1c | No | [25] |
|  | Symptom consequences | Standard care | Non-significant | 9 | High | 1c | No | [25] |
|  | Symptom controllability | Standard care | Non-significant | 9 | High | 1c | No | [25] |
|  | Overall quality of life | Standard care | Significant | 6 | Moderate | 1c | No | [26] |
|  | Physical quality of life | Standard care | Non-significant | 6 | Moderate | 1c | Yes | [26] |
|  | Cognitive quality of life | Standard care | Non-significant | 6 | Moderate | 1c | Yes | [26] |
|  | Emotional quality of life | Standard care | Non-significant | 6 | Moderate | 1c | Yes | [26] |
|  | Social quality of life | Standard care | Non-significant | 6 | Moderate | 1c | Yes | [26] |
|  | Sexual quality of life | Standard care | Non-significant | 6 | Moderate | 1c | Yes | [26] |
|  | Sexual functioning | Standard care | Non-significant | 6 | Moderate | 1c | Yes | [26] |
|  | Sexual satisfaction | Standard care | Significant | 4 | Low | 1c | No | [41] |
|  | Depression | Attention control | Non-significant | 9 | High | 1c | No | [43] |
|  | Uncertainty - ambiguity | Attention control | Significant | 9 | High | 1c | No | [43] |
|  | Symptom distress | Attention control | Significant | 9 | High | 1c | No | [43] |
|  | Overall quality of life | Attention control | Non-significant | 9 | High | 1c | No | [43] |
|  | Physical quality of life | Attention control | Significant | 9 | High | 1c | No | [43] |
|  | Cognitive quality of life | Attention control | Significant | 9 | High | 1c | No | [43] |
|  | Hospitalizations | Attention control | Non-significant | 9 | High | 1c | No | [44] |
|  | Oncology outpatient visits | Attention control | Non-significant | 9 | High | 1c | No | [44] |
|  | Emergency room visits | Attention control | Non-significant | 9 | High | 1c | No | [44] |
|  | Primary care visits | Attention control | Significant | 9 | High | 1c | No | [44] |
|  | Body image | Intervention I/intervention II | Non-significant | 7 | Moderate | 1c | No | [45] |
|  | Self-esteem | Intervention I/intervention II | Non-significant | 7 | Moderate | 1c | No | [45] |
|  | Overall quality of life | Pretest/posttest | Non-significant | 3 | Low | 4c | No | [46] |
|  | Physical quality of life | Pretest/posttest | Non-significant | 3 | Low | 4c | No | [46] |
|  | Functional quality of life | Pretest/posttest | Non-significant | 3 | Low | 4c | No | [46] |
|  | Emotional quality of life | Pretest/posttest | Significant | 3 | Low | 4c | No | [46] |
|  | Social quality of life | Pretest/posttest | Non-significant | 3 | Low | 4c | No | [46] |
|  | Quality of life - symptoms | Pretest/posttest | Non-significant | 3 | Low | 4c | No | [46] |
|  | Knowledge | Pretest/posttest | Significant | 4 | Moderate | 4c | No | [47] |
|  | Attitude | Pretest/posttest | Significant | 4 | Moderate | 4c | No | [47] |
|  | Knowledge | Intervention I/intervention II | Significant | 5 | Moderate | 4b | No | [48] |
|  | Self-care performance | Intervention I/intervention II | Non-significant | 5 | Moderate | 4b | Yes | [48] |
|  | Urinalysis | Intervention I/intervention II | Non-significant | 5 | Moderate | 4b | Yes | [48] |
|  | Urine culture | Intervention I/intervention II | Non-significant | 5 | Moderate | 4b | Yes | [48] |
| Individual and group counseling | Sexual functioning | Attention control | Non-significant | 7 | High | 1c | Yes | [42] |
|  | Uncertainty | Attention control | Partly significant | 7 | High | 1c | Yes | [42] |
|  | Anxiety/depression | Attention control | Non-significant | 7 | High | 1c | Yes | [42] |
|  | Social support | Attention control | Non-significant | 7 | High | 1c | Yes | [42] |
|  | Overall quality of life | Attention control | Non-significant | 7 | High | 1c | Yes | [42] |
|  | Physical quality of life | Attention control | Non-significant | 7 | High | 1c | Yes | [42] |
|  | Functional quality of life | Attention control | Non-significant | 7 | High | 1c | Yes | [42] |
|  | Emotional quality of life | Attention control | Non-significant | 7 | High | 1c | Yes | [42] |
|  | Social quality of life | Attention control | Non-significant | 7 | High | 1c | Yes | [42] |
| **Setting** |  |  |  |  |  |  |  |  |
| Clinic only | Sexual functioning | Attention control | Non-significant | 7 | High | 1c | Yes | [42] |
|  | Uncertainty | Attention control | Partly significant | 7 | High | 1c | Yes | [42] |
|  | Anxiety/depression | Attention control | Non-significant | 7 | High | 1c | Yes | [42] |
|  | Social support | Attention control | Non-significant | 7 | High | 1c | Yes | [42] |
|  | Overall quality of life | Attention control | Non-significant | 7 | High | 1c | Yes | [42] |
|  | Physical quality of life | Attention control | Non-significant | 7 | High | 1c | Yes | [42] |
|  | Functional quality of life | Attention control | Non-significant | 7 | High | 1c | Yes | [42] |
|  | Emotional quality of life | Attention control | Non-significant | 7 | High | 1c | Yes | [42] |
|  | Social quality of life | Attention control | Non-significant | 7 | High | 1c | Yes | [42] |
|  | Body image | Intervention I/intervention II | Non-significant | 7 | Moderate | 1c | No | [45] |
|  | Self-esteem | Intervention I/intervention II | Non-significant | 7 | Moderate | 1c | No | [45] |
|  | Knowledge | Pretest/posttest | Significant | 4 | Moderate | 4c | No | [47] |
|  | Attitude | Pretest/posttest | Significant | 4 | Moderate | 4c | No | [47] |
|  | Knowledge | Intervention I/intervention II | Significant | 5 | Moderate | 4b | No | [48] |
|  | Self-care performance | Intervention I/intervention II | Non-significant | 5 | Moderate | 4b | Yes | [48] |
|  | Urinalysis | Intervention I/intervention II | Non-significant | 5 | Moderate | 4b | Yes | [48] |
|  | Urine culture | Intervention I/intervention II | Non-significant | 5 | Moderate | 4b | Yes | [48] |
| Clinic and home | Overall quality of life | Standard care | Significant | 6 | Moderate | 1c | No | [26] |
|  | Physical quality of life | Standard care | Non-significant | 6 | Moderate | 1c | Yes | [26] |
|  | Cognitive quality of life | Standard care | Non-significant | 6 | Moderate | 1c | Yes | [26] |
|  | Emotional quality of life | Standard care | Non-significant | 6 | Moderate | 1c | Yes | [26] |
|  | Social quality of life | Standard care | Non-significant | 6 | Moderate | 1c | Yes | [26] |
|  | Sexual quality of life | Standard care | Non-significant | 6 | Moderate | 1c | Yes | [26] |
|  | Sexual functioning | Standard care | Non-significant | 6 | Moderate | 1c | Yes | [26] |
|  | Sexual satisfaction | Standard care | Significant | 4 | Low | 1c | No | [41] |
|  | Depression | Attention control | Non-significant | 9 | High | 1c | No | [43] |
|  | Uncertainty - ambiguity | Attention control | Significant | 9 | High | 1c | No | [43] |
|  | Symptom distress | Attention control | Significant | 9 | High | 1c | No | [43] |
|  | Overall quality of life | Attention control | Non-significant | 9 | High | 1c | No | [43] |
|  | Physical quality of life | Attention control | Significant | 9 | High | 1c | No | [43] |
|  | Cognitive quality of life | Attention control | Significant | 9 | High | 1c | No | [43] |
|  | Hospitalizations | Attention control | Non-significant | 9 | High | 1c | No | [44] |
|  | Oncology outpatient visits | Attention control | Non-significant | 9 | High | 1c | No | [44] |
|  | Emergency room visits | Attention control | Non-significant | 9 | High | 1c | No | [44] |
|  | Primary care visits | Attention control | Significant | 9 | High | 1c | No | [44] |
| **Materials** |  |  |  |  |  |  |  |  |
| Symptom-assessment tools | Symptom severity | Standard care | Significant | 9 | High | 1c | No | [25] |
|  | Symptom distress | Standard care | Significant | 9 | High | 1c | No | [25] |
|  | Symptom consequences | Standard care | Non-significant | 9 | High | 1c | No | [25] |
|  | Symptom controllability | Standard care | Non-significant | 9 | High | 1c | No | [25] |
|  | Depression | Attention control | Non-significant | 9 | High | 1c | No | [43] |
|  | Uncertainty - ambiguity | Attention control | Significant | 9 | High | 1c | No | [43] |
|  | Symptom distress | Attention control | Significant | 9 | High | 1c | No | [43] |
|  | Overall quality of life | Attention control | Non-significant | 9 | High | 1c | No | [43] |
|  | Physical quality of life | Attention control | Significant | 9 | High | 1c | No | [43] |
|  | Cognitive quality of life | Attention control | Significant | 9 | High | 1c | No | [43] |
|  | Hospitalizations | Attention control | Non-significant | 9 | High | 1c | No | [44] |
|  | Oncology outpatient visits | Attention control | Non-significant | 9 | High | 1c | No | [44] |
|  | Emergency room visits | Attention control | Non-significant | 9 | High | 1c | No | [44] |
|  | Primary care visits | Attention control | Significant | 9 | High | 1c | No | [44] |
|  | Overall quality of life | Pretest/posttest | Non-significant | 3 | Low | 4c | No | [46] |
|  | Physical quality of life | Pretest/posttest | Non-significant | 3 | Low | 4c | No | [46] |
|  | Functional quality of life | Pretest/posttest | Non-significant | 3 | Low | 4c | No | [46] |
|  | Emotional quality of life | Pretest/posttest | Significant | 3 | Low | 4c | No | [46] |
|  | Social quality of life | Pretest/posttest | Non-significant | 3 | Low | 4c | No | [46] |
|  | Quality of life - symptoms | Pretest/posttest | Non-significant | 3 | Low | 4c | No | [46] |
| Symptom-management guidelines | Symptom severity | Standard care | Significant | 9 | High | 1c | No | [25] |
|  | Symptom distress | Standard care | Significant | 9 | High | 1c | No | [25] |
|  | Symptom consequences | Standard care | Non-significant | 9 | High | 1c | No | [25] |
|  | Symptom controllability | Standard care | Non-significant | 9 | High | 1c | No | [25] |
|  | Sexual satisfaction | Standard care | Significant | 4 | Low | 1c | No | [41] |
|  | Depression | Attention control | Non-significant | 9 | High | 1c | No | [43] |
|  | Uncertainty - ambiguity | Attention control | Significant | 9 | High | 1c | No | [43] |
|  | Symptom distress | Attention control | Significant | 9 | High | 1c | No | [43] |
|  | Overall quality of life | Attention control | Non-significant | 9 | High | 1c | No | [43] |
|  | Physical quality of life | Attention control | Significant | 9 | High | 1c | No | [43] |
|  | Cognitive quality of life | Attention control | Significant | 9 | High | 1c | No | [43] |
|  | Hospitalizations | Attention control | Non-significant | 9 | High | 1c | No | [44] |
|  | Oncology outpatient visits | Attention control | Non-significant | 9 | High | 1c | No | [44] |
|  | Emergency room visits | Attention control | Non-significant | 9 | High | 1c | No | [44] |
|  | Primary care visits | Attention control | Significant | 9 | High | 1c | No | [44] |
|  | Body image | Intervention I/intervention II | Non-significant | 7 | Moderate | 1c | No | [45] |
|  | Self-esteem | Intervention I/intervention II | Non-significant | 7 | Moderate | 1c | No | [45] |
|  | Overall quality of life | Pretest/posttest | Non-significant | 3 | Low | 4c | No | [46] |
|  | Physical quality of life | Pretest/posttest | Non-significant | 3 | Low | 4c | No | [46] |
|  | Functional quality of life | Pretest/posttest | Non-significant | 3 | Low | 4c | No | [46] |
|  | Emotional quality of life | Pretest/posttest | Significant | 3 | Low | 4c | No | [46] |
|  | Social quality of life | Pretest/posttest | Non-significant | 3 | Low | 4c | No | [46] |
|  | Quality of life - symptoms | Pretest/posttest | Non-significant | 3 | Low | 4c | No | [46] |
| Individual care plans | Symptom severity | Standard care | Significant | 9 | High | 1c | No | [25] |
|  | Symptom distress | Standard care | Significant | 9 | High | 1c | No | [25] |
|  | Symptom consequences | Standard care | Non-significant | 9 | High | 1c | No | [25] |
|  | Symptom controllability | Standard care | Non-significant | 9 | High | 1c | No | [25] |
|  | Sexual satisfaction | Standard care | Significant | 4 | Low | 1c | No | [41] |
|  | Depression | Attention control | Non-significant | 9 | High | 1c | No | [43] |
|  | Uncertainty - ambiguity | Attention control | Significant | 9 | High | 1c | No | [43] |
|  | Symptom distress | Attention control | Significant | 9 | High | 1c | No | [43] |
|  | Overall quality of life | Attention control | Non-significant | 9 | High | 1c | No | [43] |
|  | Physical quality of life | Attention control | Significant | 9 | High | 1c | No | [43] |
|  | Cognitive quality of life | Attention control | Significant | 9 | High | 1c | No | [43] |
|  | Hospitalizations | Attention control | Non-significant | 9 | High | 1c | No | [44] |
|  | Oncology outpatient visits | Attention control | Non-significant | 9 | High | 1c | No | [44] |
|  | Emergency room visits | Attention control | Non-significant | 9 | High | 1c | No | [44] |
|  | Primary care visits | Attention control | Significant | 9 | High | 1c | No | [44] |
| Leaflets | Overall quality of life | Standard care | Significant | 6 | Moderate | 1c | No | [26] |
|  | Physical quality of life | Standard care | Non-significant | 6 | Moderate | 1c | Yes | [26] |
|  | Cognitive quality of life | Standard care | Non-significant | 6 | Moderate | 1c | Yes | [26] |
|  | Emotional quality of life | Standard care | Non-significant | 6 | Moderate | 1c | Yes | [26] |
|  | Social quality of life | Standard care | Non-significant | 6 | Moderate | 1c | Yes | [26] |
|  | Sexual quality of life | Standard care | Non-significant | 6 | Moderate | 1c | Yes | [26] |
|  | Sexual functioning | Standard care | Non-significant | 6 | Moderate | 1c | Yes | [26] |
|  | Overall quality of life | Pretest/posttest | Non-significant | 3 | Low | 4c | No | [46] |
|  | Physical quality of life | Pretest/posttest | Non-significant | 3 | Low | 4c | No | [46] |
|  | Functional quality of life | Pretest/posttest | Non-significant | 3 | Low | 4c | No | [46] |
|  | Emotional quality of life | Pretest/posttest | Significant | 3 | Low | 4c | No | [46] |
|  | Social quality of life | Pretest/posttest | Non-significant | 3 | Low | 4c | No | [46] |
|  | Quality of life - symptoms | Pretest/posttest | Non-significant | 3 | Low | 4c | No | [46] |
|  | Knowledge | Pretest/posttest | Significant | 4 | Moderate | 4c | No | [47] |
|  | Attitude | Pretest/posttest | Significant | 4 | Moderate | 4c | No | [47] |
|  | Knowledge | Intervention I/intervention II | Significant | 5 | Moderate | 4b | No | [48] |
|  | Self-care performance | Intervention I/intervention II | Non-significant | 5 | Moderate | 4b | Yes | [48] |
|  | Urinalysis | Intervention I/intervention II | Non-significant | 5 | Moderate | 4b | Yes | [48] |
|  | Urine culture | Intervention I/intervention II | Non-significant | 5 | Moderate | 4b | Yes | [48] |
| Videos | Body image | Intervention I/intervention II | Non-significant | 7 | Moderate | 1c | No | [45] |
|  | Self-esteem | Intervention I/intervention II | Non-significant | 7 | Moderate | 1c | No | [45] |
|  | Knowledge | Pretest/posttest | Significant | 4 | Moderate | 4c | No | [47] |
|  | Attitude | Pretest/posttest | Significant | 4 | Moderate | 4c | No | [47] |
| **Concepts** |  |  |  |  |  |  |  |  |
| Structured counseling only | Body image | Intervention I/intervention II | Non-significant | 7 | Moderate | 1c | No | [45] |
|  | Self-esteem | Intervention I/intervention II | Non-significant | 7 | Moderate | 1c | No | [45] |
|  | Knowledge | Intervention I/intervention II | Significant | 5 | Moderate | 4b | No | [48] |
|  | Self-care performance | Intervention I/intervention II | Non-significant | 5 | Moderate | 4b | Yes | [48] |
|  | Urinalysis | Intervention I/intervention II | Non-significant | 5 | Moderate | 4b | Yes | [48] |
|  | Urine culture | Intervention I/intervention II | Non-significant | 5 | Moderate | 4b | Yes | [48] |
| Structured and tailored counseling | Symptom severity | Standard care | Significant | 9 | High | 1c | No | [25] |
|  | Symptom distress | Standard care | Significant | 9 | High | 1c | No | [25] |
|  | Symptom consequences | Standard care | Non-significant | 9 | High | 1c | No | [25] |
|  | Symptom controllability | Standard care | Non-significant | 9 | High | 1c | No | [25] |
|  | Overall quality of life | Standard care | Significant | 6 | Moderate | 1c | No | [26] |
|  | Physical quality of life | Standard care | Non-significant | 6 | Moderate | 1c | Yes | [26] |
|  | Cognitive quality of life | Standard care | Non-significant | 6 | Moderate | 1c | Yes | [26] |
|  | Emotional quality of life | Standard care | Non-significant | 6 | Moderate | 1c | Yes | [26] |
|  | Social quality of life | Standard care | Non-significant | 6 | Moderate | 1c | Yes | [26] |
|  | Sexual quality of life | Standard care | Non-significant | 6 | Moderate | 1c | Yes | [26] |
|  | Sexual functioning | Standard care | Non-significant | 6 | Moderate | 1c | Yes | [26] |
|  | Sexual satisfaction | Standard care | Significant | 4 | Low | 1c | No | [41] |
|  | Sexual functioning | Attention control | Non-significant | 7 | High | 1c | Yes | [42] |
|  | Uncertainty | Attention control | Partly significant | 7 | High | 1c | Yes | [42] |
|  | Anxiety/depression | Attention control | Non-significant | 7 | High | 1c | Yes | [42] |
|  | Social support | Attention control | Non-significant | 7 | High | 1c | Yes | [42] |
|  | Overall quality of life | Attention control | Non-significant | 7 | High | 1c | Yes | [42] |
|  | Physical quality of life | Attention control | Non-significant | 7 | High | 1c | Yes | [42] |
|  | Functional quality of life | Attention control | Non-significant | 7 | High | 1c | Yes | [42] |
|  | Emotional quality of life | Attention control | Non-significant | 7 | High | 1c | Yes | [42] |
|  | Social quality of life | Attention control | Non-significant | 7 | High | 1c | Yes | [42] |
|  | Depression | Attention control | Non-significant | 9 | High | 1c | No | [43] |
|  | Uncertainty - ambiguity | Attention control | Significant | 9 | High | 1c | No | [43] |
|  | Symptom distress | Attention control | Significant | 9 | High | 1c | No | [43] |
|  | Overall quality of life | Attention control | Non-significant | 9 | High | 1c | No | [43] |
|  | Physical quality of life | Attention control | Significant | 9 | High | 1c | No | [43] |
|  | Cognitive quality of life | Attention control | Significant | 9 | High | 1c | No | [43] |
|  | Hospitalizations | Attention control | Non-significant | 9 | High | 1c | No | [44] |
|  | Oncology outpatient visits | Attention control | Non-significant | 9 | High | 1c | No | [44] |
|  | Emergency room visits | Attention control | Non-significant | 9 | High | 1c | No | [44] |
|  | Primary care visits | Attention control | Significant | 9 | High | 1c | No | [44] |
|  | Overall quality of life | Pretest/posttest | Non-significant | 3 | Low | 4c | No | [46] |
|  | Physical quality of life | Pretest/posttest | Non-significant | 3 | Low | 4c | No | [46] |
|  | Functional quality of life | Pretest/posttest | Non-significant | 3 | Low | 4c | No | [46] |
|  | Emotional quality of life | Pretest/posttest | Significant | 3 | Low | 4c | No | [46] |
|  | Social quality of life | Pretest/posttest | Non-significant | 3 | Low | 4c | No | [46] |
|  | Quality of life - symptoms | Pretest/posttest | Non-significant | 3 | Low | 4c | No | [46] |
|  | Knowledge | Pretest/posttest | Significant | 4 | Moderate | 4c | No | [47] |
|  | Attitude | Pretest/posttest | Significant | 4 | Moderate | 4c | No | [47] |
| Interdisciplinary orientation | Symptom severity | Standard care | Significant | 9 | High | 1c | No | [25] |
|  | Symptom distress | Standard care | Significant | 9 | High | 1c | No | [25] |
|  | Symptom consequences | Standard care | Non-significant | 9 | High | 1c | No | [25] |
|  | Symptom controllability | Standard care | Non-significant | 9 | High | 1c | No | [25] |
|  | Overall quality of life | Standard care | Significant | 6 | Moderate | 1c | No | [26] |
|  | Physical quality of life | Standard care | Non-significant | 6 | Moderate | 1c | Yes | [26] |
|  | Cognitive quality of life | Standard care | Non-significant | 6 | Moderate | 1c | Yes | [26] |
|  | Emotional quality of life | Standard care | Non-significant | 6 | Moderate | 1c | Yes | [26] |
|  | Social quality of life | Standard care | Non-significant | 6 | Moderate | 1c | Yes | [26] |
|  | Sexual quality of life | Standard care | Non-significant | 6 | Moderate | 1c | Yes | [26] |
|  | Sexual functioning | Standard care | Non-significant | 6 | Moderate | 1c | Yes | [26] |
|  | Depression | Attention control | Non-significant | 9 | High | 1c | No | [43] |
|  | Uncertainty - ambiguity | Attention control | Significant | 9 | High | 1c | No | [43] |
|  | Symptom distress | Attention control | Significant | 9 | High | 1c | No | [43] |
|  | Overall quality of life | Attention control | Non-significant | 9 | High | 1c | No | [43] |
|  | Physical quality of life | Attention control | Significant | 9 | High | 1c | No | [43] |
|  | Cognitive quality of life | Attention control | Significant | 9 | High | 1c | No | [43] |
|  | Hospitalizations | Attention control | Non-significant | 9 | High | 1c | No | [44] |
|  | Oncology outpatient visits | Attention control | Non-significant | 9 | High | 1c | No | [44] |
|  | Emergency room visits | Attention control | Non-significant | 9 | High | 1c | No | [44] |
|  | Primary care visits | Attention control | Significant | 9 | High | 1c | No | [44] |
|  | Overall quality of life | Pretest/posttest | Non-significant | 3 | Low | 4c | No | [46] |
|  | Physical quality of life | Pretest/posttest | Non-significant | 3 | Low | 4c | No | [46] |
|  | Functional quality of life | Pretest/posttest | Non-significant | 3 | Low | 4c | No | [46] |
|  | Emotional quality of life | Pretest/posttest | Significant | 3 | Low | 4c | No | [46] |
|  | Social quality of life | Pretest/posttest | Non-significant | 3 | Low | 4c | No | [46] |
|  | Quality of life - symptoms | Pretest/posttest | Non-significant | 3 | Low | 4c | No | [46] |
| Theoretical basis | Symptom severity | Standard care | Significant | 9 | High | 1c | No | [25] |
|  | Symptom distress | Standard care | Significant | 9 | High | 1c | No | [25] |
|  | Symptom consequences | Standard care | Non-significant | 9 | High | 1c | No | [25] |
|  | Symptom controllability | Standard care | Non-significant | 9 | High | 1c | No | [25] |
|  | Sexual functioning | Attention control | Non-significant | 7 | High | 1c | Yes | [42] |
|  | Uncertainty | Attention control | Partly significant | 7 | High | 1c | Yes | [42] |
|  | Anxiety/depression | Attention control | Non-significant | 7 | High | 1c | Yes | [42] |
|  | Social support | Attention control | Non-significant | 7 | High | 1c | Yes | [42] |
|  | Overall quality of life | Attention control | Non-significant | 7 | High | 1c | Yes | [42] |
|  | Physical quality of life | Attention control | Non-significant | 7 | High | 1c | Yes | [42] |
|  | Functional quality of life | Attention control | Non-significant | 7 | High | 1c | Yes | [42] |
|  | Emotional quality of life | Attention control | Non-significant | 7 | High | 1c | Yes | [42] |
|  | Social quality of life | Attention control | Non-significant | 7 | High | 1c | Yes | [42] |
|  | Anxiety/depression | Attention control | Non-significant | 7 | High | 1c | Yes | [42] |
|  | Social support | Attention control | Non-significant | 7 | High | 1c | Yes | [42] |
|  | Depression | Attention control | Non-significant | 9 | High | 1c | No | [43] |
|  | Uncertainty - ambiguity | Attention control | Significant | 9 | High | 1c | No | [43] |
|  | Symptom distress | Attention control | Significant | 9 | High | 1c | No | [43] |
|  | Overall quality of life | Attention control | Non-significant | 9 | High | 1c | No | [43] |
|  | Physical quality of life | Attention control | Significant | 9 | High | 1c | No | [43] |
|  | Cognitive quality of life | Attention control | Significant | 9 | High | 1c | No | [43] |
|  | Hospitalizations | Attention control | Non-significant | 9 | High | 1c | No | [44] |
|  | Oncology outpatient visits | Attention control | Non-significant | 9 | High | 1c | No | [44] |
|  | Emergency room visits | Attention control | Non-significant | 9 | High | 1c | No | [44] |
|  | Primary care visits | Attention control | Significant | 9 | High | 1c | No | [44] |
|  | Body image | Intervention I/intervention II | Non-significant | 7 | Moderate | 1c | No | [45] |
|  | Self-esteem | Intervention I/intervention II | Non-significant | 7 | Moderate | 1c | No | [45] |

| **Content components** | **Outcome** | **Comparison** | **Effect** | **Study quality score** | **Study quality** | **Evidence level** | **Risk type II errors** | **Reference** |
| --- | --- | --- | --- | --- | --- | --- | --- | --- |
| **Disease** |  |  |  |  |  |  |  |  |
| Etiology | Sexual functioning | Attention control | Non-significant | 7 | High | 1c | Yes | [42] |
|  | Uncertainty | Attention control | Partly significant | 7 | High | 1c | Yes | [42] |
|  | Anxiety/depression | Attention control | Non-significant | 7 | High | 1c | Yes | [42] |
|  | Social support | Attention control | Non-significant | 7 | High | 1c | Yes | [42] |
|  | Overall quality of life | Attention control | Non-significant | 7 | High | 1c | Yes | [42] |
|  | Physical quality of life | Attention control | Non-significant | 7 | High | 1c | Yes | [42] |
|  | Functional quality of life | Attention control | Non-significant | 7 | High | 1c | Yes | [42] |
|  | Emotional quality of life | Attention control | Non-significant | 7 | High | 1c | Yes | [42] |
|  | Social quality of life | Attention control | Non-significant | 7 | High | 1c | Yes | [42] |
|  | Overall quality of life | Pretest/posttest | Non-significant | 3 | Low | 4c | No | [46] |
|  | Physical quality of life | Pretest/posttest | Non-significant | 3 | Low | 4c | No | [46] |
|  | Functional quality of life | Pretest/posttest | Non-significant | 3 | Low | 4c | No | [46] |
|  | Emotional quality of life | Pretest/posttest | Significant | 3 | Low | 4c | No | [46] |
|  | Social quality of life | Pretest/posttest | Non-significant | 3 | Low | 4c | No | [46] |
|  | Quality of life - symptoms | Pretest/posttest | Non-significant | 3 | Low | 4c | No | [46] |
| Diagnostic procedures | Overall quality of life | Pretest/posttest | Non-significant | 3 | Low | 4c | No | [46] |
|  | Physical quality of life | Pretest/posttest | Non-significant | 3 | Low | 4c | No | [46] |
|  | Functional quality of life | Pretest/posttest | Non-significant | 3 | Low | 4c | No | [46] |
|  | Emotional quality of life | Pretest/posttest | Significant | 3 | Low | 4c | No | [46] |
|  | Social quality of life | Pretest/posttest | Non-significant | 3 | Low | 4c | No | [46] |
|  | Quality of life - symptoms | Pretest/posttest | Non-significant | 3 | Low | 4c | No | [46] |
| Physical symptoms | Symptom severity | Standard care | Significant | 9 | High | 1c | No | [25] |
|  | Symptom distress | Standard care | Significant | 9 | High | 1c | No | [25] |
|  | Symptom consequences | Standard care | Non-significant | 9 | High | 1c | No | [25] |
|  | Symptom controllability | Standard care | Non-significant | 9 | High | 1c | No | [25] |
|  | Sexual functioning | Attention control | Non-significant | 7 | High | 1c | Yes | [42] |
|  | Uncertainty | Attention control | Partly significant | 7 | High | 1c | Yes | [42] |
|  | Anxiety/depression | Attention control | Non-significant | 7 | High | 1c | Yes | [42] |
|  | Social support | Attention control | Non-significant | 7 | High | 1c | Yes | [42] |
|  | Overall quality of life | Attention control | Non-significant | 7 | High | 1c | Yes | [42] |
|  | Physical quality of life | Attention control | Non-significant | 7 | High | 1c | Yes | [42] |
|  | Functional quality of life | Attention control | Non-significant | 7 | High | 1c | Yes | [42] |
|  | Emotional quality of life | Attention control | Non-significant | 7 | High | 1c | Yes | [42] |
|  | Social quality of life | Attention control | Non-significant | 7 | High | 1c | Yes | [42] |
|  | Depression | Attention control | Non-significant | 9 | High | 1c | No | [43] |
|  | Uncertainty - ambiguity | Attention control | Significant | 9 | High | 1c | No | [43] |
|  | Symptom distress | Attention control | Significant | 9 | High | 1c | No | [43] |
|  | Overall quality of life | Attention control | Non-significant | 9 | High | 1c | No | [43] |
|  | Physical quality of life | Attention control | Significant | 9 | High | 1c | No | [43] |
|  | Cognitive quality of life | Attention control | Significant | 9 | High | 1c | No | [43] |
|  | Hospitalizations | Attention control | Non-significant | 9 | High | 1c | No | [44] |
|  | Oncology outpatient visits | Attention control | Non-significant | 9 | High | 1c | No | [44] |
|  | Emergency room visits | Attention control | Non-significant | 9 | High | 1c | No | [44] |
|  | Primary care visits | Attention control | Significant | 9 | High | 1c | No | [44] |
|  | Overall quality of life | Pretest/posttest | Non-significant | 3 | Low | 4c | No | [46] |
|  | Physical quality of life | Pretest/posttest | Non-significant | 3 | Low | 4c | No | [46] |
|  | Functional quality of life | Pretest/posttest | Non-significant | 3 | Low | 4c | No | [46] |
|  | Emotional quality of life | Pretest/posttest | Significant | 3 | Low | 4c | No | [46] |
|  | Social quality of life | Pretest/posttest | Non-significant | 3 | Low | 4c | No | [46] |
|  | Quality of life - symptoms | Pretest/posttest | Non-significant | 3 | Low | 4c | No | [46] |
| Psychosocial symptoms | Symptom severity | Standard care | Significant | 9 | High | 1c | No | [25] |
|  | Symptom distress | Standard care | Significant | 9 | High | 1c | No | [25] |
|  | Symptom consequences | Standard care | Non-significant | 9 | High | 1c | No | [25] |
|  | Symptom controllability | Standard care | Non-significant | 9 | High | 1c | No | [25] |
|  | Sexual satisfaction | Standard care | Significant | 4 | Low | 1c | No | [41] |
|  | Sexual functioning | Attention control | Non-significant | 7 | High | 1c | Yes | [42] |
|  | Uncertainty | Attention control | Partly significant | 7 | High | 1c | Yes | [42] |
|  | Anxiety/depression | Attention control | Non-significant | 7 | High | 1c | Yes | [42] |
|  | Social support | Attention control | Non-significant | 7 | High | 1c | Yes | [42] |
|  | Overall quality of life | Attention control | Non-significant | 7 | High | 1c | Yes | [42] |
|  | Physical quality of life | Attention control | Non-significant | 7 | High | 1c | Yes | [42] |
|  | Functional quality of life | Attention control | Non-significant | 7 | High | 1c | Yes | [42] |
|  | Emotional quality of life | Attention control | Non-significant | 7 | High | 1c | Yes | [42] |
|  | Social quality of life | Attention control | Non-significant | 7 | High | 1c | Yes | [42] |
|  | Depression | Attention control | Non-significant | 9 | High | 1c | No | [43] |
|  | Uncertainty - ambiguity | Attention control | Significant | 9 | High | 1c | No | [43] |
|  | Symptom distress | Attention control | Significant | 9 | High | 1c | No | [43] |
|  | Overall quality of life | Attention control | Non-significant | 9 | High | 1c | No | [43] |
|  | Physical quality of life | Attention control | Significant | 9 | High | 1c | No | [43] |
|  | Cognitive quality of life | Attention control | Significant | 9 | High | 1c | No | [43] |
|  | Hospitalizations | Attention control | Non-significant | 9 | High | 1c | No | [44] |
|  | Oncology outpatient visits | Attention control | Non-significant | 9 | High | 1c | No | [44] |
|  | Emergency room visits | Attention control | Non-significant | 9 | High | 1c | No | [44] |
|  | Primary care visits | Attention control | Significant | 9 | High | 1c | No | [44] |
|  | Overall quality of life | Pretest/posttest | Non-significant | 3 | Low | 4c | No | [46] |
|  | Physical quality of life | Pretest/posttest | Non-significant | 3 | Low | 4c | No | [46] |
|  | Functional quality of life | Pretest/posttest | Non-significant | 3 | Low | 4c | No | [46] |
|  | Emotional quality of life | Pretest/posttest | Significant | 3 | Low | 4c | No | [46] |
|  | Social quality of life | Pretest/posttest | Non-significant | 3 | Low | 4c | No | [46] |
|  | Quality of life - symptoms | Pretest/posttest | Non-significant | 3 | Low | 4c | No | [46] |
| **Treatment** |  |  |  |  |  |  |  |  |
| Therapeutic procedures | Overall quality of life | Standard care | Significant | 6 | Moderate | 1c | No | [26] |
|  | Physical quality of life | Standard care | Non-significant | 6 | Moderate | 1c | Yes | [26] |
|  | Cognitive quality of life | Standard care | Non-significant | 6 | Moderate | 1c | Yes | [26] |
|  | Emotional quality of life | Standard care | Non-significant | 6 | Moderate | 1c | Yes | [26] |
|  | Social quality of life | Standard care | Non-significant | 6 | Moderate | 1c | Yes | [26] |
|  | Sexual quality of life | Standard care | Non-significant | 6 | Moderate | 1c | Yes | [26] |
|  | Sexual functioning | Standard care | Non-significant | 6 | Moderate | 1c | Yes | [26] |
|  | Sexual functioning | Attention control | Non-significant | 7 | High | 1c | Yes | [42] |
|  | Uncertainty | Attention control | Partly significant | 7 | High | 1c | Yes | [42] |
|  | Anxiety/depression | Attention control | Non-significant | 7 | High | 1c | Yes | [42] |
|  | Social support | Attention control | Non-significant | 7 | High | 1c | Yes | [42] |
|  | Overall quality of life | Attention control | Non-significant | 7 | High | 1c | Yes | [42] |
|  | Physical quality of life | Attention control | Non-significant | 7 | High | 1c | Yes | [42] |
|  | Functional quality of life | Attention control | Non-significant | 7 | High | 1c | Yes | [42] |
|  | Emotional quality of life | Attention control | Non-significant | 7 | High | 1c | Yes | [42] |
|  | Social quality of life | Attention control | Non-significant | 7 | High | 1c | Yes | [42] |
| Decision-making | Depression | Attention control | Non-significant | 9 | High | 1c | No | [43] |
|  | Uncertainty - ambiguity | Attention control | Significant | 9 | High | 1c | No | [43] |
|  | Symptom distress | Attention control | Significant | 9 | High | 1c | No | [43] |
|  | Overall quality of life | Attention control | Non-significant | 9 | High | 1c | No | [43] |
|  | Physical quality of life | Attention control | Significant | 9 | High | 1c | No | [43] |
|  | Cognitive quality of life | Attention control | Significant | 9 | High | 1c | No | [43] |
|  | Hospitalizations | Attention control | Non-significant | 9 | High | 1c | No | [44] |
|  | Oncology outpatient visits | Attention control | Non-significant | 9 | High | 1c | No | [44] |
|  | Emergency room visits | Attention control | Non-significant | 9 | High | 1c | No | [44] |
|  | Primary care visits | Attention control | Significant | 9 | High | 1c | No | [44] |
| Physical symptoms | Symptom severity | Standard care | Significant | 9 | High | 1c | No | [25] |
|  | Symptom distress | Standard care | Significant | 9 | High | 1c | No | [25] |
|  | Symptom consequences | Standard care | Non-significant | 9 | High | 1c | No | [25] |
|  | Symptom controllability | Standard care | Non-significant | 9 | High | 1c | No | [25] |
|  | Sexual functioning | Attention control | Non-significant | 7 | High | 1c | Yes | [42] |
|  | Uncertainty | Attention control | Partly significant | 7 | High | 1c | Yes | [42] |
|  | Anxiety/depression | Attention control | Non-significant | 7 | High | 1c | Yes | [42] |
|  | Social support | Attention control | Non-significant | 7 | High | 1c | Yes | [42] |
|  | Overall quality of life | Attention control | Non-significant | 7 | High | 1c | Yes | [42] |
|  | Physical quality of life | Attention control | Non-significant | 7 | High | 1c | Yes | [42] |
|  | Functional quality of life | Attention control | Non-significant | 7 | High | 1c | Yes | [42] |
|  | Emotional quality of life | Attention control | Non-significant | 7 | High | 1c | Yes | [42] |
|  | Social quality of life | Attention control | Non-significant | 7 | High | 1c | Yes | [42] |
|  | Depression | Attention control | Non-significant | 9 | High | 1c | No | [43] |
|  | Uncertainty - ambiguity | Attention control | Significant | 9 | High | 1c | No | [43] |
|  | Symptom distress | Attention control | Significant | 9 | High | 1c | No | [43] |
|  | Overall quality of life | Attention control | Non-significant | 9 | High | 1c | No | [43] |
|  | Physical quality of life | Attention control | Significant | 9 | High | 1c | No | [43] |
|  | Cognitive quality of life | Attention control | Significant | 9 | High | 1c | No | [43] |
|  | Hospitalizations | Attention control | Non-significant | 9 | High | 1c | No | [44] |
|  | Oncology outpatient visits | Attention control | Non-significant | 9 | High | 1c | No | [44] |
|  | Emergency room visits | Attention control | Non-significant | 9 | High | 1c | No | [44] |
|  | Primary care visits | Attention control | Significant | 9 | High | 1c | No | [44] |
|  | Body image | Intervention I/intervention II | Non-significant | 7 | Moderate | 1c | No | [45] |
|  | Self-esteem | Intervention I/intervention II | Non-significant | 7 | Moderate | 1c | No | [45] |
|  | Overall quality of life | Pretest/posttest | Non-significant | 3 | Low | 4c | No | [46] |
|  | Physical quality of life | Pretest/posttest | Non-significant | 3 | Low | 4c | No | [46] |
|  | Functional quality of life | Pretest/posttest | Non-significant | 3 | Low | 4c | No | [46] |
|  | Emotional quality of life | Pretest/posttest | Significant | 3 | Low | 4c | No | [46] |
|  | Social quality of life | Pretest/posttest | Non-significant | 3 | Low | 4c | No | [46] |
|  | Quality of life - symptoms | Pretest/posttest | Non-significant | 3 | Low | 4c | No | [46] |
|  | Knowledge | Pretest/posttest | Significant | 4 | Moderate | 4c | No | [47] |
|  | Attitude | Pretest/posttest | Significant | 4 | Moderate | 4c | No | [47] |
|  | Knowledge | Intervention I/intervention II | Significant | 5 | Moderate | 4b | No | [48] |
|  | Self-care performance | Intervention I/intervention II | Non-significant | 5 | Moderate | 4b | Yes | [48] |
|  | Urinalysis | Intervention I/intervention II | Non-significant | 5 | Moderate | 4b | Yes | [48] |
|  | Urine culture | Intervention I/intervention II | Non-significant | 5 | Moderate | 4b | Yes | [48] |
| Psychosocial symptoms | Symptom severity | Standard care | Significant | 9 | High | 1c | No | [25] |
|  | Symptom distress | Standard care | Significant | 9 | High | 1c | No | [25] |
|  | Symptom consequences | Standard care | Non-significant | 9 | High | 1c | No | [25] |
|  | Symptom controllability | Standard care | Non-significant | 9 | High | 1c | No | [25] |
|  | Overall quality of life | Standard care | Significant | 6 | Moderate | 1c | No | [26] |
|  | Physical quality of life | Standard care | Non-significant | 6 | Moderate | 1c | Yes | [26] |
|  | Cognitive quality of life | Standard care | Non-significant | 6 | Moderate | 1c | Yes | [26] |
|  | Emotional quality of life | Standard care | Non-significant | 6 | Moderate | 1c | Yes | [26] |
|  | Social quality of life | Standard care | Non-significant | 6 | Moderate | 1c | Yes | [26] |
|  | Sexual quality of life | Standard care | Non-significant | 6 | Moderate | 1c | Yes | [26] |
|  | Sexual functioning | Standard care | Non-significant | 6 | Moderate | 1c | Yes | [26] |
|  | Sexual satisfaction | Standard care | Significant | 4 | Low | 1c | No | [41] |
|  | Sexual functioning | Attention control | Non-significant | 7 | High | 1c | Yes | [42] |
|  | Uncertainty | Attention control | Partly significant | 7 | High | 1c | Yes | [42] |
|  | Anxiety/depression | Attention control | Non-significant | 7 | High | 1c | Yes | [42] |
|  | Social support | Attention control | Non-significant | 7 | High | 1c | Yes | [42] |
|  | Overall quality of life | Attention control | Non-significant | 7 | High | 1c | Yes | [42] |
|  | Physical quality of life | Attention control | Non-significant | 7 | High | 1c | Yes | [42] |
|  | Functional quality of life | Attention control | Non-significant | 7 | High | 1c | Yes | [42] |
|  | Emotional quality of life | Attention control | Non-significant | 7 | High | 1c | Yes | [42] |
|  | Social quality of life | Attention control | Non-significant | 7 | High | 1c | Yes | [42] |
|  | Depression | Attention control | Non-significant | 9 | High | 1c | No | [43] |
|  | Uncertainty - ambiguity | Attention control | Significant | 9 | High | 1c | No | [43] |
|  | Symptom distress | Attention control | Significant | 9 | High | 1c | No | [43] |
|  | Overall quality of life | Attention control | Non-significant | 9 | High | 1c | No | [43] |
|  | Physical quality of life | Attention control | Significant | 9 | High | 1c | No | [43] |
|  | Cognitive quality of life | Attention control | Significant | 9 | High | 1c | No | [43] |
|  | Hospitalizations | Attention control | Non-significant | 9 | High | 1c | No | [44] |
|  | Oncology outpatient visits | Attention control | Non-significant | 9 | High | 1c | No | [44] |
|  | Emergency room visits | Attention control | Non-significant | 9 | High | 1c | No | [44] |
|  | Primary care visits | Attention control | Significant | 9 | High | 1c | No | [44] |
|  | Overall quality of life | Pretest/posttest | Non-significant | 3 | Low | 4c | No | [46] |
|  | Physical quality of life | Pretest/posttest | Non-significant | 3 | Low | 4c | No | [46] |
|  | Functional quality of life | Pretest/posttest | Non-significant | 3 | Low | 4c | No | [46] |
|  | Emotional quality of life | Pretest/posttest | Significant | 3 | Low | 4c | No | [46] |
|  | Social quality of life | Pretest/posttest | Non-significant | 3 | Low | 4c | No | [46] |
|  | Quality of life - symptoms | Pretest/posttest | Non-significant | 3 | Low | 4c | No | [46] |
| **Symptom-management** |  |  |  |  |  |  |  |  |
| Symptom etiology | Symptom severity | Standard care | Significant | 9 | High | 1c | No | [25] |
|  | Symptom distress | Standard care | Significant | 9 | High | 1c | No | [25] |
|  | Symptom consequences | Standard care | Non-significant | 9 | High | 1c | No | [25] |
|  | Symptom controllability | Standard care | Non-significant | 9 | High | 1c | No | [25] |
|  | Overall quality of life | Standard care | Significant | 6 | Moderate | 1c | No | [26] |
|  | Physical quality of life | Standard care | Non-significant | 6 | Moderate | 1c | Yes | [26] |
|  | Cognitive quality of life | Standard care | Non-significant | 6 | Moderate | 1c | Yes | [26] |
|  | Emotional quality of life | Standard care | Non-significant | 6 | Moderate | 1c | Yes | [26] |
|  | Social quality of life | Standard care | Non-significant | 6 | Moderate | 1c | Yes | [26] |
|  | Sexual quality of life | Standard care | Non-significant | 6 | Moderate | 1c | Yes | [26] |
|  | Sexual functioning | Standard care | Non-significant | 6 | Moderate | 1c | Yes | [26] |
|  | Sexual satisfaction | Standard care | Significant | 4 | Low | 1c | No | [41] |
|  | Depression | Attention control | Non-significant | 9 | High | 1c | No | [43] |
|  | Uncertainty - ambiguity | Attention control | Significant | 9 | High | 1c | No | [43] |
|  | Symptom distress | Attention control | Significant | 9 | High | 1c | No | [43] |
|  | Overall quality of life | Attention control | Non-significant | 9 | High | 1c | No | [43] |
|  | Physical quality of life | Attention control | Significant | 9 | High | 1c | No | [43] |
|  | Cognitive quality of life | Attention control | Significant | 9 | High | 1c | No | [43] |
|  | Hospitalizations | Attention control | Non-significant | 9 | High | 1c | No | [44] |
|  | Oncology outpatient visits | Attention control | Non-significant | 9 | High | 1c | No | [44] |
|  | Emergency room visits | Attention control | Non-significant | 9 | High | 1c | No | [44] |
|  | Primary care visits | Attention control | Significant | 9 | High | 1c | No | [44] |
|  | Body image | Intervention I/intervention II | Non-significant | 7 | Moderate | 1c | No | [45] |
|  | Self-esteem | Intervention I/intervention II | Non-significant | 7 | Moderate | 1c | No | [45] |
|  | Overall quality of life | Pretest/posttest | Non-significant | 3 | Low | 4c | No | [46] |
|  | Physical quality of life | Pretest/posttest | Non-significant | 3 | Low | 4c | No | [46] |
|  | Functional quality of life | Pretest/posttest | Non-significant | 3 | Low | 4c | No | [46] |
|  | Emotional quality of life | Pretest/posttest | Significant | 3 | Low | 4c | No | [46] |
|  | Social quality of life | Pretest/posttest | Non-significant | 3 | Low | 4c | No | [46] |
|  | Quality of life - symptoms | Pretest/posttest | Non-significant | 3 | Low | 4c | No | [46] |
|  | Knowledge | Pretest/posttest | Significant | 4 | Moderate | 4c | No | [47] |
|  | Attitude | Pretest/posttest | Significant | 4 | Moderate | 4c | No | [47] |
| Symptom assessment | Symptom severity | Standard care | Significant | 9 | High | 1c | No | [25] |
|  | Symptom distress | Standard care | Significant | 9 | High | 1c | No | [25] |
|  | Symptom consequences | Standard care | Non-significant | 9 | High | 1c | No | [25] |
|  | Symptom controllability | Standard care | Non-significant | 9 | High | 1c | No | [25] |
|  | Depression | Attention control | Non-significant | 9 | High | 1c | No | [43] |
|  | Uncertainty - ambiguity | Attention control | Significant | 9 | High | 1c | No | [43] |
|  | Symptom distress | Attention control | Significant | 9 | High | 1c | No | [43] |
|  | Overall quality of life | Attention control | Non-significant | 9 | High | 1c | No | [43] |
|  | Physical quality of life | Attention control | Significant | 9 | High | 1c | No | [43] |
|  | Cognitive quality of life | Attention control | Significant | 9 | High | 1c | No | [43] |
|  | Hospitalizations | Attention control | Non-significant | 9 | High | 1c | No | [44] |
|  | Oncology outpatient visits | Attention control | Non-significant | 9 | High | 1c | No | [44] |
|  | Emergency room visits | Attention control | Non-significant | 9 | High | 1c | No | [44] |
|  | Primary care visits | Attention control | Significant | 9 | High | 1c | No | [44] |
| Goal-setting and planning | Symptom severity | Standard care | Significant | 9 | High | 1c | No | [25] |
|  | Symptom distress | Standard care | Significant | 9 | High | 1c | No | [25] |
|  | Symptom consequences | Standard care | Non-significant | 9 | High | 1c | No | [25] |
|  | Symptom controllability | Standard care | Non-significant | 9 | High | 1c | No | [25] |
|  | Depression | Attention control | Non-significant | 9 | High | 1c | No | [43] |
|  | Uncertainty - ambiguity | Attention control | Significant | 9 | High | 1c | No | [43] |
|  | Symptom distress | Attention control | Significant | 9 | High | 1c | No | [43] |
|  | Overall quality of life | Attention control | Non-significant | 9 | High | 1c | No | [43] |
|  | Physical quality of life | Attention control | Significant | 9 | High | 1c | No | [43] |
|  | Cognitive quality of life | Attention control | Significant | 9 | High | 1c | No | [43] |
|  | Hospitalizations | Attention control | Non-significant | 9 | High | 1c | No | [44] |
|  | Oncology outpatient visits | Attention control | Non-significant | 9 | High | 1c | No | [44] |
|  | Emergency room visits | Attention control | Non-significant | 9 | High | 1c | No | [44] |
|  | Primary care visits | Attention control | Significant | 9 | High | 1c | No | [44] |
| Symptom prevention | Symptom severity | Standard care | Significant | 9 | High | 1c | No | [25] |
|  | Symptom distress | Standard care | Significant | 9 | High | 1c | No | [25] |
|  | Symptom consequences | Standard care | Non-significant | 9 | High | 1c | No | [25] |
|  | Symptom controllability | Standard care | Non-significant | 9 | High | 1c | No | [25] |
|  | Overall quality of life | Standard care | Significant | 6 | Moderate | 1c | No | [26] |
|  | Physical quality of life | Standard care | Non-significant | 6 | Moderate | 1c | Yes | [26] |
|  | Cognitive quality of life | Standard care | Non-significant | 6 | Moderate | 1c | Yes | [26] |
|  | Emotional quality of life | Standard care | Non-significant | 6 | Moderate | 1c | Yes | [26] |
|  | Social quality of life | Standard care | Non-significant | 6 | Moderate | 1c | Yes | [26] |
|  | Sexual quality of life | Standard care | Non-significant | 6 | Moderate | 1c | Yes | [26] |
|  | Sexual functioning | Standard care | Non-significant | 6 | Moderate | 1c | Yes | [26] |
|  | Sexual satisfaction | Standard care | Significant | 4 | Low | 1c | No | [41] |
|  | Sexual functioning | Attention control | Non-significant | 7 | High | 1c | Yes | [42] |
|  | Uncertainty | Attention control | Partly significant | 7 | High | 1c | Yes | [42] |
|  | Anxiety/depression | Attention control | Non-significant | 7 | High | 1c | Yes | [42] |
|  | Social support | Attention control | Non-significant | 7 | High | 1c | Yes | [42] |
|  | Overall quality of life | Attention control | Non-significant | 7 | High | 1c | Yes | [42] |
|  | Physical quality of life | Attention control | Non-significant | 7 | High | 1c | Yes | [42] |
|  | Functional quality of life | Attention control | Non-significant | 7 | High | 1c | Yes | [42] |
|  | Emotional quality of life | Attention control | Non-significant | 7 | High | 1c | Yes | [42] |
|  | Social quality of life | Attention control | Non-significant | 7 | High | 1c | Yes | [42] |
|  | Depression | Attention control | Non-significant | 9 | High | 1c | No | [43] |
|  | Uncertainty - ambiguity | Attention control | Significant | 9 | High | 1c | No | [43] |
|  | Symptom distress | Attention control | Significant | 9 | High | 1c | No | [43] |
|  | Overall quality of life | Attention control | Non-significant | 9 | High | 1c | No | [43] |
|  | Physical quality of life | Attention control | Significant | 9 | High | 1c | No | [43] |
|  | Cognitive quality of life | Attention control | Significant | 9 | High | 1c | No | [43] |
|  | Hospitalizations | Attention control | Non-significant | 9 | High | 1c | No | [44] |
|  | Oncology outpatient visits | Attention control | Non-significant | 9 | High | 1c | No | [44] |
|  | Emergency room visits | Attention control | Non-significant | 9 | High | 1c | No | [44] |
|  | Primary care visits | Attention control | Significant | 9 | High | 1c | No | [44] |
|  | Knowledge | Pretest/posttest | Significant | 4 | Moderate | 4c | No | [47] |
|  | Attitude | Pretest/posttest | Significant | 4 | Moderate | 4c | No | [47] |
|  | Knowledge | Intervention I/intervention II | Significant | 5 | Moderate | 4b | No | [48] |
|  | Self-care performance | Intervention I/intervention II | Non-significant | 5 | Moderate | 4b | Yes | [48] |
|  | Urinalysis | Intervention I/intervention II | Non-significant | 5 | Moderate | 4b | Yes | [48] |
|  | Urine culture | Intervention I/intervention II | Non-significant | 5 | Moderate | 4b | Yes | [48] |
| Symptom treatment | Symptom severity | Standard care | Significant | 9 | High | 1c | No | [25] |
|  | Symptom distress | Standard care | Significant | 9 | High | 1c | No | [25] |
|  | Symptom consequences | Standard care | Non-significant | 9 | High | 1c | No | [25] |
|  | Symptom controllability | Standard care | Non-significant | 9 | High | 1c | No | [25] |
|  | Overall quality of life | Standard care | Significant | 6 | Moderate | 1c | No | [26] |
|  | Physical quality of life | Standard care | Non-significant | 6 | Moderate | 1c | Yes | [26] |
|  | Cognitive quality of life | Standard care | Non-significant | 6 | Moderate | 1c | Yes | [26] |
|  | Emotional quality of life | Standard care | Non-significant | 6 | Moderate | 1c | Yes | [26] |
|  | Social quality of life | Standard care | Non-significant | 6 | Moderate | 1c | Yes | [26] |
|  | Sexual quality of life | Standard care | Non-significant | 6 | Moderate | 1c | Yes | [26] |
|  | Sexual functioning | Standard care | Non-significant | 6 | Moderate | 1c | Yes | [26] |
|  | Sexual satisfaction | Standard care | Significant | 4 | Low | 1c | No | [41] |
|  | Sexual functioning | Attention control | Non-significant | 7 | High | 1c | Yes | [42] |
|  | Uncertainty | Attention control | Partly significant | 7 | High | 1c | Yes | [42] |
|  | Anxiety/depression | Attention control | Non-significant | 7 | High | 1c | Yes | [42] |
|  | Social support | Attention control | Non-significant | 7 | High | 1c | Yes | [42] |
|  | Overall quality of life | Attention control | Non-significant | 7 | High | 1c | Yes | [42] |
|  | Physical quality of life | Attention control | Non-significant | 7 | High | 1c | Yes | [42] |
|  | Functional quality of life | Attention control | Non-significant | 7 | High | 1c | Yes | [42] |
|  | Emotional quality of life | Attention control | Non-significant | 7 | High | 1c | Yes | [42] |
|  | Social quality of life | Attention control | Non-significant | 7 | High | 1c | Yes | [42] |
|  | Depression | Attention control | Non-significant | 9 | High | 1c | No | [43] |
|  | Uncertainty - ambiguity | Attention control | Significant | 9 | High | 1c | No | [43] |
|  | Symptom distress | Attention control | Significant | 9 | High | 1c | No | [43] |
|  | Overall quality of life | Attention control | Non-significant | 9 | High | 1c | No | [43] |
|  | Physical quality of life | Attention control | Significant | 9 | High | 1c | No | [43] |
|  | Cognitive quality of life | Attention control | Significant | 9 | High | 1c | No | [43] |
|  | Hospitalizations | Attention control | Non-significant | 9 | High | 1c | No | [44] |
|  | Oncology outpatient visits | Attention control | Non-significant | 9 | High | 1c | No | [44] |
|  | Emergency room visits | Attention control | Non-significant | 9 | High | 1c | No | [44] |
|  | Primary care visits | Attention control | Significant | 9 | High | 1c | No | [44] |
|  | Body image | Intervention I/intervention II | Non-significant | 7 | Moderate | 1c | No | [45] |
|  | Self-esteem | Intervention I/intervention II | Non-significant | 7 | Moderate | 1c | No | [45] |
|  | Overall quality of life | Pretest/posttest | Non-significant | 3 | Low | 4c | No | [46] |
|  | Physical quality of life | Pretest/posttest | Non-significant | 3 | Low | 4c | No | [46] |
|  | Functional quality of life | Pretest/posttest | Non-significant | 3 | Low | 4c | No | [46] |
|  | Emotional quality of life | Pretest/posttest | Significant | 3 | Low | 4c | No | [46] |
|  | Social quality of life | Pretest/posttest | Non-significant | 3 | Low | 4c | No | [46] |
|  | Quality of life - symptoms | Pretest/posttest | Non-significant | 3 | Low | 4c | No | [46] |
|  | Knowledge | Pretest/posttest | Significant | 4 | Moderate | 4c | No | [47] |
|  | Attitude | Pretest/posttest | Significant | 4 | Moderate | 4c | No | [47] |
|  | Knowledge | Intervention I/intervention II | Significant | 5 | Moderate | 4b | No | [48] |
|  | Self-care performance | Intervention I/intervention II | Non-significant | 5 | Moderate | 4b | Yes | [48] |
|  | Urinalysis | Intervention I/intervention II | Non-significant | 5 | Moderate | 4b | Yes | [48] |
|  | Urine culture | Intervention I/intervention II | Non-significant | 5 | Moderate | 4b | Yes | [48] |
| Evaluation and modification of symptom management strategies | Symptom severity | Standard care | Significant | 9 | High | 1c | No | [25] |
|  | Symptom distress | Standard care | Significant | 9 | High | 1c | No | [25] |
|  | Symptom consequences | Standard care | Non-significant | 9 | High | 1c | No | [25] |
|  | Symptom controllability | Standard care | Non-significant | 9 | High | 1c | No | [25] |
|  | Depression | Attention control | Non-significant | 9 | High | 1c | No | [43] |
|  | Uncertainty - ambiguity | Attention control | Significant | 9 | High | 1c | No | [43] |
|  | Symptom distress | Attention control | Significant | 9 | High | 1c | No | [43] |
|  | Overall quality of life | Attention control | Non-significant | 9 | High | 1c | No | [43] |
|  | Physical quality of life | Attention control | Significant | 9 | High | 1c | No | [43] |
|  | Cognitive quality of life | Attention control | Significant | 9 | High | 1c | No | [43] |
|  | Hospitalizations | Attention control | Non-significant | 9 | High | 1c | No | [44] |
|  | Oncology outpatient visits | Attention control | Non-significant | 9 | High | 1c | No | [44] |
|  | Emergency room visits | Attention control | Non-significant | 9 | High | 1c | No | [44] |
|  | Primary care visits | Attention control | Significant | 9 | High | 1c | No | [44] |
|  | Knowledge | Intervention I/intervention II | Significant | 5 | Moderate | 4b | No | [48] |
|  | Self-care performance | Intervention I/intervention II | Non-significant | 5 | Moderate | 4b | Yes | [48] |
|  | Urinalysis | Intervention I/intervention II | Non-significant | 5 | Moderate | 4b | Yes | [48] |
|  | Urine culture | Intervention I/intervention II | Non-significant | 5 | Moderate | 4b | Yes | [48] |
| **Resources** |  |  |  |  |  |  |  |  |
| Personal capabilities | Symptom severity | Standard care | Significant | 9 | High | 1c | No | [25] |
|  | Symptom distress | Standard care | Significant | 9 | High | 1c | No | [25] |
|  | Symptom consequences | Standard care | Non-significant | 9 | High | 1c | No | [25] |
|  | Symptom controllability | Standard care | Non-significant | 9 | High | 1c | No | [25] |
|  | Overall quality of life | Standard care | Significant | 6 | Moderate | 1c | No | [26] |
|  | Physical quality of life | Standard care | Non-significant | 6 | Moderate | 1c | Yes | [26] |
|  | Cognitive quality of life | Standard care | Non-significant | 6 | Moderate | 1c | Yes | [26] |
|  | Emotional quality of life | Standard care | Non-significant | 6 | Moderate | 1c | Yes | [26] |
|  | Social quality of life | Standard care | Non-significant | 6 | Moderate | 1c | Yes | [26] |
|  | Sexual quality of life | Standard care | Non-significant | 6 | Moderate | 1c | Yes | [26] |
|  | Sexual functioning | Standard care | Non-significant | 6 | Moderate | 1c | Yes | [26] |
|  | Sexual functioning | Attention control | Non-significant | 7 | High | 1c | Yes | [42] |
|  | Uncertainty | Attention control | Partly significant | 7 | High | 1c | Yes | [42] |
|  | Anxiety/depression | Attention control | Non-significant | 7 | High | 1c | Yes | [42] |
|  | Social support | Attention control | Non-significant | 7 | High | 1c | Yes | [42] |
|  | Overall quality of life | Attention control | Non-significant | 7 | High | 1c | Yes | [42] |
|  | Physical quality of life | Attention control | Non-significant | 7 | High | 1c | Yes | [42] |
|  | Functional quality of life | Attention control | Non-significant | 7 | High | 1c | Yes | [42] |
|  | Emotional quality of life | Attention control | Non-significant | 7 | High | 1c | Yes | [42] |
|  | Social quality of life | Attention control | Non-significant | 7 | High | 1c | Yes | [42] |
|  | Sexual satisfaction | Standard care | Significant | 4 | Low | 1c | No | [41] |
| Social network | Overall quality of life | Standard care | Significant | 6 | Moderate | 1c | No | [26] |
|  | Physical quality of life | Standard care | Non-significant | 6 | Moderate | 1c | Yes | [26] |
|  | Cognitive quality of life | Standard care | Non-significant | 6 | Moderate | 1c | Yes | [26] |
|  | Emotional quality of life | Standard care | Non-significant | 6 | Moderate | 1c | Yes | [26] |
|  | Social quality of life | Standard care | Non-significant | 6 | Moderate | 1c | Yes | [26] |
|  | Sexual quality of life | Standard care | Non-significant | 6 | Moderate | 1c | Yes | [26] |
|  | Sexual functioning | Standard care | Non-significant | 6 | Moderate | 1c | Yes | [26] |
|  | Sexual satisfaction | Standard care | Significant | 4 | Low | 1c | No | [41] |
|  | Sexual functioning | Attention control | Non-significant | 7 | High | 1c | Yes | [42] |
|  | Uncertainty | Attention control | Partly significant | 7 | High | 1c | Yes | [42] |
|  | Anxiety/depression | Attention control | Non-significant | 7 | High | 1c | Yes | [42] |
|  | Social support | Attention control | Non-significant | 7 | High | 1c | Yes | [42] |
|  | Overall quality of life | Attention control | Non-significant | 7 | High | 1c | Yes | [42] |
|  | Physical quality of life | Attention control | Non-significant | 7 | High | 1c | Yes | [42] |
|  | Functional quality of life | Attention control | Non-significant | 7 | High | 1c | Yes | [42] |
|  | Emotional quality of life | Attention control | Non-significant | 7 | High | 1c | Yes | [42] |
|  | Social quality of life | Attention control | Non-significant | 7 | High | 1c | Yes | [42] |
|  | Anxiety/depression | Attention control | Non-significant | 7 | High | 1c | Yes | [42] |
|  | Social support | Attention control | Non-significant | 7 | High | 1c | Yes | [42] |
|  | Depression | Attention control | Non-significant | 9 | High | 1c | No | [43] |
|  | Uncertainty - ambiguity | Attention control | Significant | 9 | High | 1c | No | [43] |
|  | Symptom distress | Attention control | Significant | 9 | High | 1c | No | [43] |
|  | Overall quality of life | Attention control | Non-significant | 9 | High | 1c | No | [43] |
|  | Physical quality of life | Attention control | Significant | 9 | High | 1c | No | [43] |
|  | Cognitive quality of life | Attention control | Significant | 9 | High | 1c | No | [43] |
|  | Hospitalizations | Attention control | Non-significant | 9 | High | 1c | No | [44] |
|  | Oncology outpatient visits | Attention control | Non-significant | 9 | High | 1c | No | [44] |
|  | Emergency room visits | Attention control | Non-significant | 9 | High | 1c | No | [44] |
|  | Primary care visits | Attention control | Significant | 9 | High | 1c | No | [44] |
|  | Overall quality of life | Pretest/posttest | Non-significant | 3 | Low | 4c | No | [46] |
|  | Physical quality of life | Pretest/posttest | Non-significant | 3 | Low | 4c | No | [46] |
|  | Functional quality of life | Pretest/posttest | Non-significant | 3 | Low | 4c | No | [46] |
|  | Emotional quality of life | Pretest/posttest | Significant | 3 | Low | 4c | No | [46] |
|  | Social quality of life | Pretest/posttest | Non-significant | 3 | Low | 4c | No | [46] |
|  | Quality of life - symptoms | Pretest/posttest | Non-significant | 3 | Low | 4c | No | [46] |
| Healthcare services | Symptom severity | Standard care | Significant | 9 | High | 1c | No | [25] |
|  | Symptom distress | Standard care | Significant | 9 | High | 1c | No | [25] |
|  | Symptom consequences | Standard care | Non-significant | 9 | High | 1c | No | [25] |
|  | Symptom controllability | Standard care | Non-significant | 9 | High | 1c | No | [25] |
|  | Overall quality of life | Standard care | Significant | 6 | Moderate | 1c | No | [26] |
|  | Physical quality of life | Standard care | Non-significant | 6 | Moderate | 1c | Yes | [26] |
|  | Cognitive quality of life | Standard care | Non-significant | 6 | Moderate | 1c | Yes | [26] |
|  | Emotional quality of life | Standard care | Non-significant | 6 | Moderate | 1c | Yes | [26] |
|  | Social quality of life | Standard care | Non-significant | 6 | Moderate | 1c | Yes | [26] |
|  | Sexual quality of life | Standard care | Non-significant | 6 | Moderate | 1c | Yes | [26] |
|  | Sexual functioning | Standard care | Non-significant | 6 | Moderate | 1c | Yes | [26] |
|  | Sexual functioning | Attention control | Non-significant | 7 | High | 1c | Yes | [42] |
|  | Uncertainty | Attention control | Partly significant | 7 | High | 1c | Yes | [42] |
|  | Anxiety/depression | Attention control | Non-significant | 7 | High | 1c | Yes | [42] |
|  | Social support | Attention control | Non-significant | 7 | High | 1c | Yes | [42] |
|  | Overall quality of life | Attention control | Non-significant | 7 | High | 1c | Yes | [42] |
|  | Physical quality of life | Attention control | Non-significant | 7 | High | 1c | Yes | [42] |
|  | Functional quality of life | Attention control | Non-significant | 7 | High | 1c | Yes | [42] |
|  | Emotional quality of life | Attention control | Non-significant | 7 | High | 1c | Yes | [42] |
|  | Social quality of life | Attention control | Non-significant | 7 | High | 1c | Yes | [42] |
|  | Anxiety/depression | Attention control | Non-significant | 7 | High | 1c | Yes | [42] |
|  | Social support | Attention control | Non-significant | 7 | High | 1c | Yes | [42] |
|  | Depression | Attention control | Non-significant | 9 | High | 1c | No | [43] |
|  | Uncertainty - ambiguity | Attention control | Significant | 9 | High | 1c | No | [43] |
|  | Symptom distress | Attention control | Significant | 9 | High | 1c | No | [43] |
|  | Overall quality of life | Attention control | Non-significant | 9 | High | 1c | No | [43] |
|  | Physical quality of life | Attention control | Significant | 9 | High | 1c | No | [43] |
|  | Cognitive quality of life | Attention control | Significant | 9 | High | 1c | No | [43] |
|  | Hospitalizations | Attention control | Non-significant | 9 | High | 1c | No | [44] |
|  | Oncology outpatient visits | Attention control | Non-significant | 9 | High | 1c | No | [44] |
|  | Emergency room visits | Attention control | Non-significant | 9 | High | 1c | No | [44] |
|  | Primary care visits | Attention control | Significant | 9 | High | 1c | No | [44] |
|  | Body image | Intervention I/intervention II | Non-significant | 7 | Moderate | 1c | No | [45] |
|  | Self-esteem | Intervention I/intervention II | Non-significant | 7 | Moderate | 1c | No | [45] |
|  | Overall quality of life | Pretest/posttest | Non-significant | 3 | Low | 4c | No | [46] |
|  | Physical quality of life | Pretest/posttest | Non-significant | 3 | Low | 4c | No | [46] |
|  | Functional quality of life | Pretest/posttest | Non-significant | 3 | Low | 4c | No | [46] |
|  | Emotional quality of life | Pretest/posttest | Significant | 3 | Low | 4c | No | [46] |
|  | Social quality of life | Pretest/posttest | Non-significant | 3 | Low | 4c | No | [46] |
|  | Quality of life - symptoms | Pretest/posttest | Non-significant | 3 | Low | 4c | No | [46] |
